# The unmet supportive care needs of people affected by cancer during the COVID-19 pandemic: an integrative review

**DOI:** 10.1007/s11764-022-01275-z

**Published:** 2022-10-29

**Authors:** H. Legge, K. Toohey, P. S. Kavanagh, C. Paterson

**Affiliations:** 1 Faculty of Health, University of Canberra, Bruce, ACT Australia; 2 Prehabilitation, Activity, Cancer, Exercise and Survivorship (PACES) Research Group, University of Canberra, Bruce, ACT 2601 Australia; 3 Justice and Society, University of South Australia, Adelaide, SA Australia; 4 Robert Gordon University, Aberdeen, Scotland, UK; 5 Canberra Health Services, SYNERGY Nursing & Midwifery Research Centre | ACT Health Directorate, Level 3, Building 6, Canberra Hospital | GPO Box 825, Canberra, ACT 2601 Australia

**Keywords:** Supportive care, Cancer, Unmet needs, COVID-19, Integrative review, Evidence synthesis

## Abstract

**Purpose:**

To critically synthesise evidence regarding the supportive care needs of those living with cancer during the COVID-19 pandemic.

**Methods:**

An integrative systematic review followed a pre-registered protocol, reported according to the Preferred Reporting Items for Systematic Reviews and Meta-analysis (PRISMA) Guidelines. We searched three databases (CINAHL, MEDLINE, and APA PsycINFO) using keywords and included all qualitative, quantitative, and mixed methods studies irrespective of research design published between December 2019 and February 2022. All articles were double screened according to a pre-determined eligibility criterion with reference lists of the final included studies checked for further studies. The review process was managed using Covidence systematic review software. Data from the studies were extracted, methodological quality appraisal conducted, and a narrative synthesis conducted.

**Results:**

Eighteen publications were included. The findings identified that individuals affected by cancer reported a range of physical, psychological, social, and health system unmet needs during the global pandemic. Unique to the pandemic itself, there was fear of the unknown of the longer-term impact that the pandemic would have on treatment outcomes, cancer care follow-up, and clinical service delays.

**Conclusion:**

Many individuals living with cancer experienced unmet needs and distress throughout the different waves of the COVID-19 pandemic, irrespective of cancer type, stage, and demographic factors.

**Implications for Cancer Survivors:**

We recommend clinicians use these findings to identify the individual person-centred needs to optimise recovery as we transition to the post-pandemic cancer care.

**Supplementary Information:**

The online version contains supplementary material available at 10.1007/s11764-022-01275-z.

## Introduction

Globally, cancer is a significant cause of mortality. In 2020, cancer caused ten million deaths, equating to nearly 1 in 6 people [1]. Many individuals affected by cancer require timely supportive care and rehabilitation in routine clinical service delivery [1]. In March 2020, the World Health Organization (WHO) declared COVID-19 as a global pandemic [2]. This pandemic has consumed peoples’ existing way of life in every aspect, from lockdowns, mandated mask wearing, to transitioning new models of cancer care delivery [3], and adhering to ever changing social distancing requirements [4]. Cancer services had to manage burgeoning clinical service demand due to COVID-19 and adapt to adhere to constant changes in legislation, restrictions, and transition cancer service models of care to models of telehealth [5].

Supportive care is defined as a person-centred holistic approach to the provision of cancer services for those living with or affected by cancer [3, 6]. This holistic lens to care encompasses the informational, social, psychological, spiritual, and physical needs during diagnosis, treatment, or follow-up phases, including issues of health promotion and prevention, survivorship, palliation, and bereavement [7]. An existing suite of systematic reviews have already identified the unmet supportive care needs in a variety of cancer groups [7–12] prior to the COVID-19 pandemic underscoring a range of unmet supportive care needs for people affected by cancer. Noteworthy, one recent systematic review [12] excluded patients reporting unmet supportive care needs during the COVID-19 pandemic because this was beyond the scope of their review. Therefore, evidence is yet to be pooled and critically synthesised to enable health care professionals and researchers to comprehensively understand the impacts of COVID-19 on supportive care experiences among people affected by cancer [3]. Gaining new insights about the supportive care needs experienced during the COVID-19 pandemic will not only inform transitions of care moving into the endemic but may highlight helpful knowledge insights which could be applied to learnings in the preparation for future pandemics within cancer health care systems and care needs.

An interruption or change to the accessibility of cancer screening, diagnostic, and treatment services has a significantly negative impact on the outcomes of cancer treatment for patients [13]. It has been suggested that addressing supportive care needs is the foundation of a successful intervention because of its positive influence on quality of life and psychosocial outcomes [6, 7, 10]. Therefore, the purpose of this systematic review was to critically synthesise evidence related to supportive care needs of those affected by cancer during the COVID-19 pandemic and address the research question: “What are the unmet supportive care needs among people diagnosed with cancer during the COVID-19 pandemic?”.

## Methods

This integrative systematic review [14] is reported according to the Preferred Reporting Items for Systematic Reviews and Meta-Analyses (PRIMSA) guidelines [15]. We pre-registered the integrative review protocol on the National Institute for Health Research PROSPERO – International Prospective Register of systematic reviews with the review available from: https://www.crd.york.ac.uk/prospero/display_record.php?RecordID=313525.

## Pre-selection eligibility criteria

### Types of studies

All qualitative, quantitative, and mixed methods studies irrespective of research design which identified supportive care needs among people affected by cancer during the COVID-19 pandemic. The WHO declared the first case of the Novel Coronavirus on the 31^st^ December 2019 [16] and later declared COVID-19 as pandemic on the 11^th^ March 2020 [2]. Therefore, the database search was limited to December 2019 to February 2022 to capture all studies related to the research question. All commentaries, editorials, and studies where supportive care needs were not reported and published in non-English language were excluded.

### Types of participants

All participants affected by cancer irrespective of age, gender, cancer type, stage, or treatment and their family members and caregivers were included.

### Types of outcome measures

The primary outcome of this review was related to unmet supportive care needs (e.g. the Supportive Care Needs Survey [17]) and qualitative experiences, informed by the definition of supportive care [7] explicitly reported in relation to the COVID-19 pandemic (see Table 1).

**Table 1:**
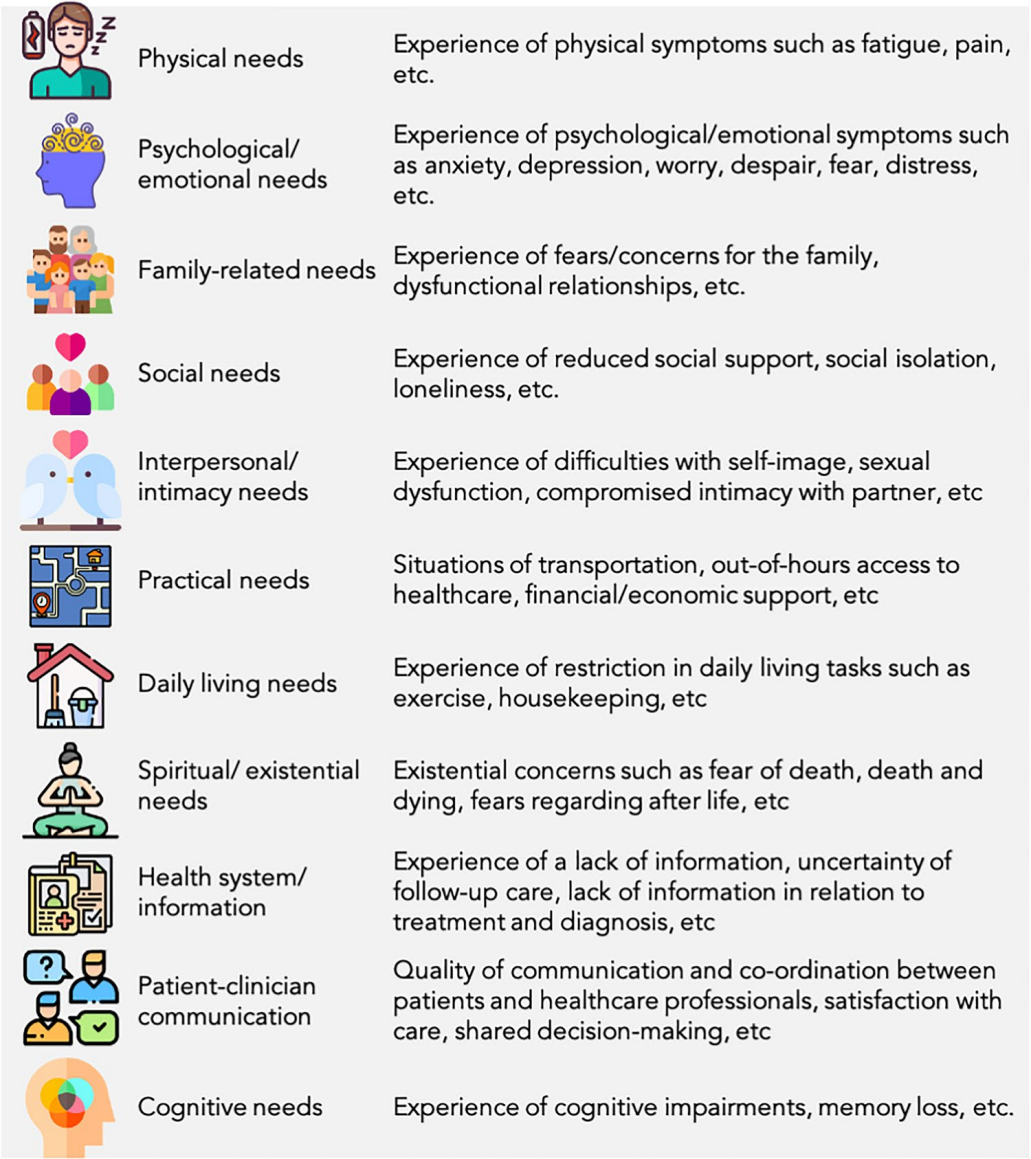
Classification of supportive care needs

## Literature search

We searched MEDLINE, CINAHL, and PsychINFO using a wide range of keywords and free text items to increase the sensitivity and inclusiveness (see Supplementary Table 1). The electronic searches began on 24^th^ February 2022 and concluded on 25^th^ February 2022. All records were managed using the software package Endnote X20 and uploaded to Covidence systematic review software with duplicate records were removed and pre-selection eligibility criterion applied to all records.

## Study selection

Two authors (HL, CP) independently reviewed the publications (titles and abstracts) applying the eligibility criteria. One author (HL) retrieved all full-text articles that met inclusion criteria and all authors independently screening the full-text articles with disagreements resolved through consensus discussion.

## Data extraction and management

Data extraction was performed on the retained full-text studies meeting the inclusion criteria. The data were extracted by one reviewer (HL) and independently quality checked by a second reviewer (CP and PK). The data extraction tables were developed and tested on a small sample of studies and then further refined through discussion among the reviewers. The first table of data extraction included information on the purpose, setting, country, sample size, participant characteristics, sampling used, response rate, attrition, design, time points, and data collection tools. The second data extraction table related to the supportive care needs outcome data according to the classification of supportive care needs.

## Quality appraisal

The quality appraisal of all included studies was conducted by utilising the Mixed Methods Appraisal Tool (MMAT) [18]. The MMAT enabled quality appraisal of qualitative research, randomised controlled trials, quantitative descriptive studies, and mixed methods studies. There are seven questions for each category of study design, ranked as “Yes” (green), “Unclear” (yellow), or “No” (red). The quality appraisal enabled the research team to identify limitations and potential bias within each of the individual studies. No study was excluded based upon individual methodological quality appraisal scores to enable an understanding of the current state of the evidence base.

## Data synthesis

This review completed tabulation of primary research studies and use of narrative synthesis to generate findings. The data synthesis process followed the integrated review methodology proposed by Whittemore and Knafl [14]. Data synthesis involved data reduction (subgroup classification by study design and domain of unmet need, with results tabulated), data comparison (identifying patterns and themes through clustering and counting and making contrasts and comparisons), and conclusion drawing and verification (synthesis of subgroup analysis to inform a comprehensive understanding of the topic, verified with the primary source data for accuracy).

## Results

**Fig. 1:**
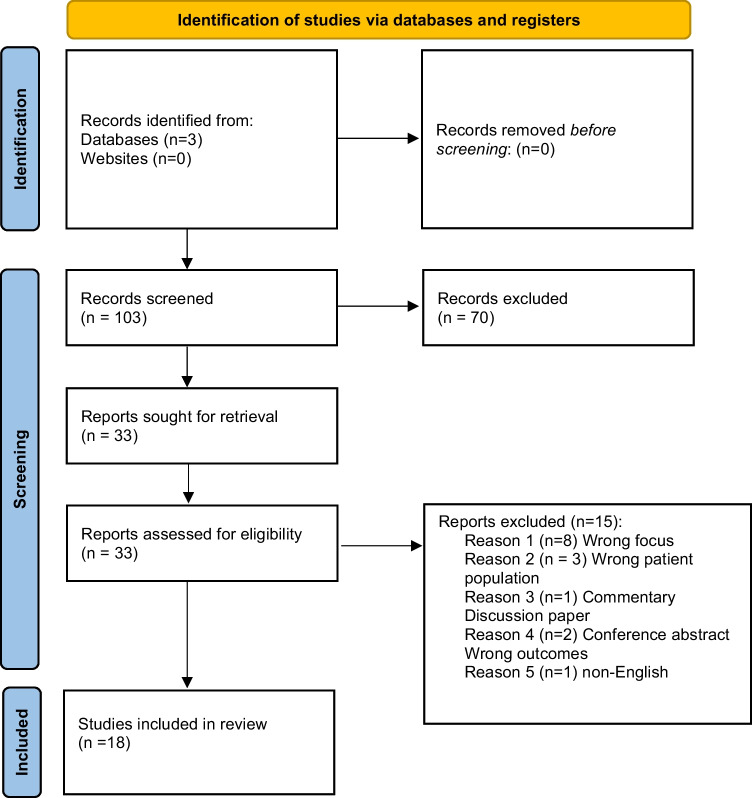
PRSIMA diagram. From: Page MJ, McKenzie JE, Bossuyt PM, Boutron I, Hoffmann TC, Mulrow CD, et al. The PRISMA 2020 statement: an updated guideline for reporting systematic reviews. BMJ 2021;372:n71. https://doi.org/10.1136/bmj.n71

Of the 122 publications retrieved from the search, we removed 19 duplicates (see Fig. 1). In total, 33 papers were reviewed in full-text and 18 papers included. There were a range of study designs underscoring that this is a developing evidence base; studies included five qualitative, 12 quantitative, and one mixed methods study. Across the included studies, there was a range of methodological quality (see Table 2), with the methodological quality overall considered good across the included studies. Studies were conducted in a range of countries, including USA (*n* = 7), Germany (*n* = 1), UK (*n* = 3), Ireland (*n* = 1), Italy (*n* = 1), Canada (*n* = 1), Iran (*n* = 1), Brazil (*n* = 1), Turkey (*n* = 1), and Australia (*n* = 1). Sample size varied from 16 to 1529 participants, with a total sample size of 3924 represented in the systematic review. Cancer type varied throughout the studies (neurological: *n* = 2; haematological: *n* = 3; head and neck cancers: *n* = 4; breast cancer: *n* = 2; varied cancer types: *n* = 4; and unreported cancer types: *n* = 3). Population across the studies varied from children and families (*n* = 3), adults (*n* = 8), older adults (*n* = 5), and unreported (*n* = 2) (see Table 3).

**Table 2:**
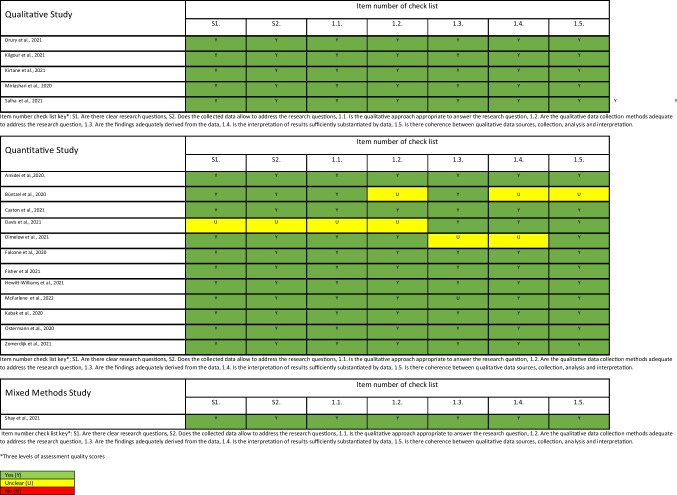
Quality assessment of included studies

**Table 3:** Overview of included studies

Author and year Country	Aim	Setting	Sample size	Participants	Sampling	Response rate	Attrition	Design	Time points	Data collection tools
Amidei et al., 2020 Country: USA	To identify the impact of the coronavirus pandemic on brain tumour charities and not-for-profit organisations that support the brain tumour community	Different International Brain Tumour Organisations from around the world	*N* = 77	Brain tumour organisations	Convenience	59%	NA	Categorical and qualitative survey	1	Questionnaires: An anonymous online 37 – question survey. Ran from May 6^th^ 2020 to June 1^st^ 2020 Responses were grouped into three categories, (1) Organisational characteristics (2) The impact of COVID-19 on services provided by brain tumour patient organisations (3) The impact of COVID-19 on financial and human resources for brain tumour patient organisations. Descriptive statistics were used to analyse categorical questions. Content thematic analysis was used to extract themes from the qualitative data obtained from the open-ended questions. Text responses were reviewed by 3 reviewers to assure themes were accurately captured
Büntzel et al., 2020 Country: Germany	To learn more about experiences of German patients affected by cancer during the pandemic	Germany Oncology Services System Südharz Hospital, Franziskus Hospital, University Hospital	*N* = 433	The median age of our participants was between 50 and 60 years The cancer types most often reported were head and neck cancer (*n* = 92) and breast cancer (*n* = 69) 138 patients were still under treatment and 227 were survivors. Another 15 patients were in palliative situation. 53 have not reported their actual tumour status	Convenience	Not reported	NA	Categorical and qualitative survey	1	Questionnaires: 4 Anonymous Online Surveys were distributed to the Oncology Services across several German Hospitals to patients, physicians, other caregivers and psycho-oncologists and spiritual care givers. Between 16/04/2020 and 15/05/2020. Responses were split into Focus Groups based on the population Metric Data were only assessed concerning Federal State (physician, medical staff, patients, and psychologists), cancer entity (patients), and whether physicians, staff, psychologists were (1) mainly involved in care of in and or out-patients and (2) in contact with patients suffering from COVID-19 Scale questions were used to assess the impact and consequences of the German Measures of COVID-19 management for patients Simple or multiple-choice questions were used to determine the influence of COVID-19 on the treating physician’s life Allegory of a thermometer was used for one question to capture the current emotional stress of the treating medical staff Free Answers: Psychologists/spiritual care givers had the possibility to add free answers for selected items
Caston et al., 2021 Country: USA	Evaluate the association between fear of COVID-19, mental health outcomes, and delays in care delivery for underserved patients with cancer during the pandemic	Nationwide Online survey distributed by Patient Advocate Foundation	*N* = 1529	Breast, Gastro-intestinal, Genitourinary, Gynaecological, Haematology, Other Over 50% of population was aged between 56 and 75 Co-morbidities were self-reported 0- cancer only 1–2- + co-morbidity 3 + co-morbidity	Convenience	*N* = 1199 78%	1^st^ survey: 1199 eligible responses 2^nd^ survey: 448 (29%)	Observational longitudinal study	2 (1) 20^th^ May–11^th^ July 2020 (2) 2^nd^–23^rd^ December	Surveys: were distributed via email to those who received PAF services from July 2019 to 2020; individuals received three reminder emails. Surveys contained questions that focused on individual experiences with COVID-19 and the psychological, emotional, physical, and material effects from the pandemic COVID-19 diagnosis was self-reported for either themselves, a household member, or both in either the first or second survey COVID-19 Specific Characteristics: perceived risk of COVID-19 was determined using the weekly average of COVID-19 cases per 100,000 for each respondent’s county the week prior to survey submission Fear of COVID-19 Scale (FCV-19S): validated using the Hospital Anxiety and Depression Scale and the Perceived Vulnerability to Disease Scale Psychological Distress (outcome): determined using a four-item questionnaire by Holingue and colleagues assessing psychological symptoms for the past 7 days Delayed Care (outcome): question specific to whether care had been delayed or interrupted due to the COVID-19 pandemic. Answers were categorised into (1) patient election, (2) hospital or provider election, (3) income loss, (4) insurance loss, or (5) difficulty accessing medications or other medical care. Delayed care was dichotomised as any versus none for modelling
Davis et al., 2021 Country: USA	To explore oncology out-patients implementation of a standardised telephone outreach service during COVID-19	Comprehensive Cancer Care Centre (RHLCCC) of North-western Medicine	*N* = 63	Over 50% of participants were aged 60–80 Cancer type unreported	Convenience	Not clearly reported	NA	Service evaluation	1	Patients with qualifying ICD-10 codes listed in their problem list or visit diagnosis were sent PROMs 72 h prior to an appointment in the cancer centre. Patients then completed the PROMs via personal computer, tablet, or smart phone. The assessment included depression, anxiety, fatigue, pain interference, and physical function, along with checklists of practical and psychosocial care needs. Patients receive the questionnaire no more frequently than once a month and can opt out
Dimelow et al., 2021 Country: UK	To explore perceptions of fear of cancer and fear of COVID-19 and to report patient preference for follow-up consultation in HNC survivors	Online survey	*N* = 103	Previously treated head and neck cancer patients were eligible 64 males, 39 females Largest number of participants were over 75 (36/103)	Convenience	56%	NA	Categorical and qualitative survey	1	Clinical information retrieved from the hospital patient record system was anonymised and categorised as age (< 55, 55–64, 65–74,75 or over), gender (male, female), clinical stage (early T1N0/T2N0, or late), site (oral, oropharyngeal, laryngeal, other), osteoradionecrosis (Y/N), surgery (Y/N), free flap (non, soft, composite), radiotherapy (Y/N), chemotherapy (Y/N), and time since primary diagnosis (< 12 months, 12–23, 24–59, 60 months, or more) A postal survey was undertaken in the 2 weeks post-consultation, including fear of recurrence
Drury et al., 2021 Country: Ireland	Explored experiences of cancer care in Ireland during the COVID-19 pandemic	Ireland during the COVID-19 pandemic	*N* = 16	Melanoma, breast, prostate, lung, colon cancers	Convenience sampling via social media	100%	Not reported	Longitudinal descriptive qualitative study	3 in total This paper refers to 1	Interviews: Interviews were guided by a semi-structured interview schedule exploring participants’ perceptions of changes in the continuity of care, perceived risks/benefits of new methods of care delivery, information needs, and worries about cancer relating to COVID-19. Both surveys provided a 5-point Likert Scale response: 1.The national comprehensive Cancer Network Distress Thermometer (NCCN-DT): is a validated self-reported screening tool to measure psychosocial distress in cancer settings 2.The two item Connor-Davidson Resilience Scale (CD-RISC2): evaluates participants resilience via two items; (1) able to adapt to change and (2) tend to bounce back after illness or hardship All interviews were conducted by a single researcher. Interviews were conducted by telephone (*n* = 15) and Microsoft teams (*n* = 1) and were recorded and transcribed verbatim to assist with analysis. Field notes were recorded immediately after each interview to support analysis and research reflexivity After T1 interviews, all participants received a copy of their interview transcript and were provided with an opportunity to amend or clarify their responses prior to data analysis. To enhance the depth of analysis and validation of the study findings, participants will receive a summary of the thematic analysis of interviews conducted at the previous timepoint
Falcone et al., 2020 Country: Italy	To explore the outbreak’s impact on their emotional well-being and quality of life	Endocrine Cancer Centre, Rome Italy	*N* = 137	Males = 30 Females = 40 Mean age = 57 Papillary thyroid cancer = 40 Follicular thyroid cancer = 5 Poorly differentiated thyroid cancer = 5 Medullary thyroid cancer = 15 Anaplastic thyroid cancer = 1 Adrenal cancer = 3 NSCLC = 1	Convenience	Survey 1 *n* = 70 (51%) Survey 2 *N* = 61 (44.5%)	NA	Categorical and qualitative survey	1	COVID-19 Patient Impact Survey- 21 item questionnaire designed ad hoc by their team to explore and measure the emotional/overall impact of the rapidly escalating COVID-19 pandemic in Italy a.Included a 6 item Core Component designed to explore and quantify the outbreaks emotional impact on the cancer patients b.Mandatory responses c.Produced a COVID-19 Concern Score (0–12) Validated Italian Translation of the European Organisation for Research and Treatment of Cancer Quality of Life Questionnaire. Consisted of 30 items representing global health/ quality of life, five function subscales (physical, emotional, role, cognitive and social) Scored in accordance with EORTC QLQ-C30 scoring manual
Fisher et al. 2021 Country: USA	Examined pandemic on psychosocial functioning of adolescent and emerging adult survivors and their parents	Neuro-oncology survivor clinic	*N* = 122	55 participating families 44 survivors 48 parents The sample of survivors was 55% male, 84.2% White, and on average 19.62 years old at the time of participation Parents were mostly mothers (*n* = 43, 89.6%) and 45.8% had at least a bachelor’s degree)	Convenience	55/122 45.1%	NA	Categorical and qualitative survey	1	1.Survivors completed the Environmental Influences on Child Health Outcomes COVID-19 child self-reported form which assessed pandemic impacts on their psychosocial functioning. Included their ability to received health care services and their social connectedness relative to before the pandemic. 20 item questionnaire measured how satisfied survivors were with their life before and during the pandemic and well as the perceive impact of COVID-19 on survivors 2.Parents completed the COVID-19 Exposure and Family Impact Survey which measured disruptions in families lives due to the COVID-19 pandemic via 25 yes/no questions. The questionnaire also assessed the impact of COVID-19 on family functioning, the caregiver’s ability to parent and the parents’ mental health, using a 4-point Likert scale Parents completed a demographic form, and the survivor’s tumour and treatment characteristics were abstracted from chart review to generate their Neurological Predictive Scale (NPS) score
Hulbert-Williams et al., 2021 Country: UK	To investigate the impact on unmet needs and psychosocial well-being via measuring participants unmet supportive care needs, psychological distress, and quality of life	UK-based Maggie’s Cancer Centres	*N* = 144 *N* = 41 (2019) *N* = 103 (2020)	77% were female Included people with cancer and their support networks were recruited. The most prevalent diagnosis was breast cancer 41 were recruited pre-pandemic and 103 participants were recruited during the COVID-19 pandemic	Convenience	Unreported due to the nature of advertisement via social media	NA	Prospectively planned, cross-sectional study	2 1 pre-pandemic June/July 2019 2 during pandemic June/July 2020	Questionnaire hosted by JISC Online Surveys. Participants were recruited by an on-site researcher and via social media advertisements Patient unmet needs were assess using the short form of the Supportive Care Needs Survey, a 34-item Support Network unmet needs were assessed using the closely related Supportive Care Needs Survey, a 44-item measure of four domains of unmet needs Depression, Anxiety and Stress were assessed in both groups using the 21 item DASS Patients QOL was assessed using the Functional Assessment of Cancer Therapy- General: a 33-item assessment oh physical, social/ family, emotional, and functional cancer-related well-being over the previous 7-day period QOL in support network participants: Caregivers Oncology QOL questionnaire; 29 item measure indicates ten dimensions of QOL including psychological well-being; burden; relationship with health care; administration and finance; coping; physical well-being; self-esteem; leisure time; social support; and private life
Kilgour et al., 2021 Country: Canada	To understand the needs and experiences of older adult cancer survivors across COVID-19	Cancer Centre of South-eastern Ontario	*N* = 24	Ages ranged from 63 to 83 years old with slightly more participants who identified as female (*n* = 14) and male (*n* = 10) All participants had been discharged from cancer care within the last 12 months and had been diagnosed with Breast(*n* = 12) or colorectal cancer (*n* = 12)	Convenience	Survey 1 *n* = 24. Survey 2 *n* = 18 and Survey 3 *n* = 18 75% response rate	6	Longitudinal descriptive qualitative study	3 (1) July/August 2020, (2) January/February 2021, and (3) March 2021	Conducted individual, semi-structured qualitative interviews. Authors completed telephone interviews with participants at three time points during the pandemic. Interviews lasted approximately 45 min, were audio recorded and transcribed verbatim by a professional transcription agency During the third interview, an infographic of the preliminary study findings created with the assistance of plain language editors. The infographic was used to discuss the interpretation of results from the first two data collection time points and elicit further perspectives Interview questions touched on a range of topics, including cancer-related concerns, coping, health challenges, and changes to health care appointment delivery. In this article, they focus on the cancer and health service delivery-related data and findings
Kirtane et al., 2021 Country: USA	To characterise the lived experiences of LA-HNC patients and their health care providers during the COVID-19 pandemic	NCI-designated comprehensive cancer centre during the COVID-19 pandemic	*N* = 33 20 = patients and 13 = HNC providers	Average age was 60.6 years old Cancer diagnosis: Oropharynx = 14 Nasopharynx = 3 Oral cavity = 1 Supraglottic Larynx = 1 Ethmoid sinus tumour = 1	Convenience	Not reported	NA	Categorical and qualitative Survey	1	After providing verbal informed consent, patients participated in individual interviews via telephone or zoom that were audio recorded and averaged 75 min in length. Patients also self-reported their demographic and clinical characteristics via an online survey using REDCap. And clinical data were confirmed via electronic medical record review Semi-structured interviews were informed by an interpretive approach, aiming to understand patient and provider experiences, perspectives, and meaning-making processes. Guides included questions and exploratory probes about treatment experiences during the COVID-19 pandemic, which is the focus of tis paper Patient interviews were conducted by trained study coordinators who were unknown to participants. Coordinator training was led by a qualitative specialist and included conducting a pilot interview with one patient each
McFarlane et al., 2022 Country: UK	To describe the care of hospitalised cancer patients dying with COVID-19	Tertiary referral cancer centre	N = 34	Aged between 64 and 76 *N* = 16 males and *n* = 18 females Breast = 7 GI = 5 Gynaecological = 4 Head and neck = 4 Lymphoma = 1 Melanoma = 2 Other haematological = 5 Renal = 1 Thoracic = 6 Urology = 1 Other = 2	Convenience	Wave 1 (25^th^ March 2020–1^st^ May 2020) *n* = 19 and Wave 2 (5^th^ December 2020–1^st^ March 2021) *n* = 15	29 non-ventilated COVID-19 PR positive patients died during the defined period. 5 patients with strong suspicion for COVID-19 also died	Retrospective study using thematic analysis	2	Data was extracted from medication charts and electronic patient records by clinicians using a standardised proforma. Including patient demographics and risk factors for COVID-19 infection such as age and gender Data was also collected to describe the main domains of palliative and end of life care service provision: Identification of individual patient needs- described using the validated integrated palliative outcome scale (IPOS)-17 IPOS items scored 0–4 with higher scores representing increased severity of symptoms/concerns Management of pain and other symptoms: prescriptions for medicators across the four main end of life symptom domains of pain, anxiety, nausea, and respiratory secretions Communicator and decision-making: Documentation of advance care planning discussions, including evidence of anticipatory care planning such as treatment escalation plans and DNACPR orders were described Responsiveness of the palliative care team: referrals and reason for palliative care referral, number of times the patients was reviewed by the palliative care team, time from onset of COVID-19 symptoms to initial palliative care review and time from first palliative care review to death were described
Mirlashari et al., 2020 Country: Iran	To investigate the perspectives of children with cancer and their families in this era of the COVID-19 pandemic	Paediatric Hospital in Tehran, Iran	*N* = 21	4 female children 13 mothers,1 father and 3 paediatric oncology nurses ALL = 13 AML = 1 Ewing’s Sarcoma = 1 Lymphoma = 1	Purposive sampling	100%	NA	Categorical and qualitative survey using thematic analysis	1	Telephone interviews were conducted on participants. The sampling continued until sufficient knowledge about the research questions was obtained. Data collection stopped when data saturation was reached The participants were selected based on the researcher's previous knowledge of families with children living with cancer. The objectives of the study were explained to each participant, and they were well informed that interview would be recorded. If they agreed, the meetings were then scheduled. Interviews were semi-structured, using interview guidance
Ostermann et al., 2020 Country: USA	To explore risk stratification in haematology/oncology patients during the COVID-19 pandemic	University of North Carolina health care system	*N* = 286 were called	Not reported	Convenience	245 = 86% successful reached via phone	NA	Cross-sectional	1	Risk Stratification: Patients were risk stratified using a general medical health composite score (HCS) and a cancer-specific risk (CSR) stratification based on disease and treatment characteristics. The correlation between HCS and CSR was measured using Spearman’s correlation. It includes in-patient admissions and ER visits in the past year, active medications, unique providers seen in the past year, high-impact chronic conditions, uncontrolled chronic diseases, in-patient psychiatry admission in the past 5 years, age, insurance, and primary care provider status. Each component is weighted to provide a total score of 0–100, with patients categorised as low (0–8), moderate (9–20), or high risk (21–100) Outreach: A multi-disciplinary team developed a focused needs assessment script with recommended interventions for patients categorised as high-risk by either method. The number of patient needs identified and referrals for services made in the first month of outreach are reported. The template outlined interventions for identified needs, including referral to social work, palliative care, and the patient and family resource centre, which includes staff and volunteers to provide emotional support and direct patients to additional resources. It concluded with a summary of active issues, interventions made, and time frame for follow-up
Salha et al., 2021 Country: Brazil	Understand individual demands and experiences during the COVID-19 pandemic Guiding question: who are you in the COVID-19 pandemic?	Caregivers who are registered users of a centralised state drug supple service in Goiania, Goias, brazil	*N* = 42	Female = 23 Male = 19 Majority aged 31–60	Convenience	Not reported	NA	Qualitative phenomenological study	1	The data collection instrument was created by the researchers in the form of a questionnaire and included a sociodemographic assessment with eight questions on the familial relationship with the user of the oncological drug, age, gender, education, religion, marital status, number of people living in the home, and family income, in addition to five semi-structured questions that addressed self-care (the practice of physical activity, type of activity, frequency of exercise, and place where exercise is practised), concerns (financial and emotional), perceptions (physical and emotional health), and attitudes (compliance with health rules and what care practices they perform) of informal caregivers in the face of the COVID-19 pandemic
Shay et al., 2021 Country: USA	Explored adolescent and young adult cancer survivor experiences during COVID-19	University of Texas health science centre	*N* = 39 AYA’s completed the survey and 24 AYAs also participated in the focus groups	Young adult cancer patients, survivors, and caregivers. All AYA were aged 18–39 with a history of a cancer diagnosis regardless of time from diagnosis or treatment status	Convenience	39 surveys 24 focus group	N/A	Categorical and qualitative survey with content analysis	2	Semi-structured focus group guide. The guide was used to assess the impact of COVID-19 in the early stages of the pandemic, how AYA’s responded, issues related to cancer care and access and barriers and coping strategies and resources during the ongoing pandemic Descriptive statistics: were used to summarise sample characteristics and proportions impacted by specific factors related to COVID-19 Thematic analysis: to examine focus group data. Themes and categories are obtained from the data on the context of the pre-determined questions
Yildiz et al., 2020 Country: Turkey	To assess supportive care needs, compliance with home exercise program, quality of life level, and anxiety level during the COVID-19	Hacettepe University Turkey	*N* = 108	62% were male, average age was 50 MM = 36 Lymphoma = 36 Leukaemia = 26 MDS = 3	Convenience	101 93%	NA	Categorical and qualitative survey	1	Supportive Care Needs: were assessed via the Supportive Care Needs Survey-Short form; inquiries about 4 different aspects of supportive care needs including health care and information, daily life, sexuality, and psychological needs Compliance with exercise program: interviewed over the phone regarding their compliance with exercise program. Assigned to the patients post performance measures and prescribed based on the ACSM cancer guidelines. Weekly compliance was recorded Quality of Life: The European Cancer Research and Treatment Organisation QOL Questionnaire- Cancer30: including 30 items which are scored between 1 and 4. Consisting of three sub-headings; general health, functionality, and symptoms Anxiety level: The state-trait anxiety inventory was used to determine anxiety level of the participants
Zomerdijk et al., 2021 Country: Australia	Identify the psychological impacts of the COVID-19 pandemic on haematology patients	Community groups (Leukaemia Foundation), professional member societies and working groups (Victorian COVID-19 and Cancer Network) and Clinical Trial Groups (Australian Leukaemia and Lymphoma Group)	*N* = 394	Age not reported Gender not reported Leukaemia = 107 Lymphoma = 136 Myeloma = 73 Other haematological cancers = 78	Convenience	Not reported	NA	Qualitative online cross-sectional survey	1	Demographics: collected on individuals information including age, gender, postcode, marital status, education level, employment status and number of dependents living in the home during COVID-19. Medical characteristics: regarding primary diagnoses, treatment, and disease state. Cancer care experience: 4 questions designed by the research team to better understand the care experiences of respondents during the pandemic. Financial concerns: two items were designed to explore respondents’ financial well-being during the pandemic. Perceived risk and impact of COVID-19 on cancer management: 5 questions were designed to investigate respondents concerns about the impact of COVID-19 on their own health and their perceived risk of contracting COVID-19 Psychological distress: measured using the Kessler 10—item assessment Unmet supportive care needs: captured using the Subdomains Health Systema and Information needs (11 items) and Patient Care and Support needs (5 items) of the short form Supportive Care needs Survey Fear of Cancer recurrence: 9 item Severity Subscale of the Fear of Cancer Recurrence Inventory

### Experiences of unmet supportive care needs

There were a range of unmet supportive care needs related to physical, psychological/emotional, cognitive, patient-clinician communication, health system/information, spiritual, daily living, interpersonal/intimacy, practical, family, and social needs (see Tables 4 and 5 for an overview). Detailed information in relation to the unmet supportive care needs of the included studies is in Supplementary Tables 2 and 3.

**Table 4:** Overview of supportive care needs explored in quantitative studies

Author and year	Physical needs	Psychological/emotional needs	Cognitive needs	Patient-clinician communication needs	Health system/information needs	Spiritual needs	Daily living needs	Interpersonal/intimacy needs	Practical needs	Family-related needs	Social needs	Number of domains explored in each study
Amidei et al., 2020	**✓**	**✓**	-	**✓**	**✓**	**✓**	**✓**	-	**✓**	**✓**	**✓**	9
Büntzel et al., 2020	**✓**	**✓**	-	-	**✓**	-	-	-	**✓**	-	**✓**	5
Caston et al., 2021	-	**✓**	-	-	-	-	-	-	**✓**	-	**✓**	3
Davis et al., 2021	**✓**	**✓**	-	-	-	-	**✓**	-	-	-	-	3
Dimelow et al., 2021	-	**✓**	-	-	-	-	-	-	-	-	-	1
Falcone et al., 2020	-	**✓**	-	-	-	-	-	-	**✓**	-	**✓**	3
Fisher et al., 2021	**✓**	**✓**	-	-	-	-	-	-	**✓**	**✓**	**✓**	5
Hulburt-Williams et al., 2021	-	**✓**	-	**✓**	**✓**	-	-	**✓**	-	**✓**	**✓**	6
McFarlene et al., 2022	**✓**	**✓**	-	-	**✓**	**✓**	-	-	**✓**	**✓**	-	6
Shay et al., 2021	**✓**	**✓**	-	**✓**	**✓**	-	**✓**	-	**✓**	-	**✓**	7
Kabak. et al., 2020	**✓**	**✓**	-	-	-	-	-	-	**✓**	-	-	3
Ostermann et. al., 2020	**✓**	**✓**	-	**✓**	**✓**	-	-	-	**✓**	-	-	5
Zomerdijk et al., 2021	-	**✓**	-	-	**✓**	-	-	-	**✓**	**✓**	-	4

**Table 5:** Overview of supportive care needs explored in qualitative studies

Categories	Themes within a category	Findings	Synthesised findings
Anxiety and distress	About catching COVID-19	F12	Anxiety and distress regarding catching COVID-19 due to recognising this population are at an increased infection risk due to cancer diagnosis How such feelings of anxiety led to individuals isolating themselves from their support systems to protect themselves and their family or being isolated by regulations imposed by governments causing disruptions to attendance to hospital appointments The overall effect this had on their psychosocial well-being
	About the unknown of COVID-19	F16	
	About attending the hospital	F2 F4	
Infection control	At an increased risk of COVID-19	F1	
	Complying with the health orders	F24	
	Developing strategies to mitigate ‘corona phobia’	F17	
Social isolation	When attending appointments	F2, F4, F10, F14	
	To avoid catching COVID-19	F13, F26	
	Lack of peer and social support	F7, F32	
Access to health care	Telehealth and decreased person-centred care	F6, F9	Patient-clinician communication and health system information disruptions due to transition to telehealth
	Restricted opportunities to engage with health care professionals and decreased quality of care	F5, F29, F11, F15, F27	
	Confusion around health orders	F20, F29	
Sacrifices	Sacrificing self-care due to work and financial demands	F22, F23	Daily living needs and practical needs related to work and financial demands
	Family needs sacrificed because of emphasis into COVID-19 Prevention	F19	
Positives	Increased Family time and a new perspective on life	F8	New perspectives on life due to increase time with loved ones, applying previous learned coping strategies
	Resilience from previous cancer diagnosis	F31	
	Coping strategies from previous cancer diagnosis	F30	
	Positive infection control measures in hospital	F3	

### Physical needs

The physical needs were commonly reported across the included studies as a well-being concern. Physical needs were well described in the palliative care cancer participants [19]. Common needs reported included fatigue, weakness, and mobility issues affecting physical functioning and activities of daily living because of restrictions during the COVID-19 pandemic [19–23]. People affected by cancer were socially isolated due to the implications of the COVID-19 pandemic. Many experienced exacerbations of additional onset of pain [19, 21, 24] because of a reduction to mobility which made accessing an already restricted health care service significantly more difficult. Furthermore, the negative effect of gastro-intestinal symptomatology of nausea, vomiting, poor appetite, a dry mouth, or constipation were also expressed [19] compounding the nutritional deficits experienced by those affected by cancer as a daily unmet need during the pandemic [21]. Lastly, participants noted that the COVID-19 pandemic negatively affected their physical well-being through disturbances to sleep, eating, and exercise [22].

### Psychological needs

Many of the participants irrespective of age, cancer type, or treatment regimen expressed unmet needs related to psychological or emotional domains of well-being [19–21, 24–28]. The fear of contracting COVID-19 in people affected by cancer was further compounded due to their own recognition that they were at an increased risk of infection which led to anxiety and distress.

It contributes to a little bit of anxiety because, you hear over and over and over, people that are in a higher risk category. And, you know, by you being diagnosed with cancer, you’re definitely put to the top of that list [29] page 4.

Children and their families also expressed fear of the unknown in relation to COVID-19. Many participants described that they had come to terms with living with cancer, but were afraid, experienced anxiety and a sense of a lack of control due to the ongoing pandemic as illustrated by this quote:

corona is strange and unknown to me [30] page 3.

Many participants commented they were always wearing a mask and went to extreme levels in cleaning and sanitising; and for some individuals even showering after leaving and returning to their house [29]. People affected by cancer acknowledged that COVID-19 was frightening for everyone, not just for those affected by cancer [29]. Noteworthy, some individuals reported that an additional diagnosis of cancer during the pandemic further compounded a sense of an additional “phobia” [30]. Many participants expressed to only feeling safe and secure when at home [30] but when they left the house this caused significant heightened levels of anxiety. People living with cancer voiced [23] issues due to the public perception that they were not visibly ill, and therefore lacked insight into the additional infection control measures needed to protect people with cancer, including those who had immunocompromised status*.* Strategies driven by fear of contracting COVID-19 [31, 32] included taking extra precautionary measures to avoid people, places, hospitals, and for some even family members all of which had an emotional impact. Participants also expressed fear that staff in their local hospitals would not be wearing adequate personal protective equipment (PPE) to keep them safe and protected from the virus while receiving care and treatment [25]. There was also fear of the unknown of the long-term impact that the pandemic would have on treatment outcomes, care follow-up, clinical service delays, and the overall impact that this would have on them in the future [23, 24, 26]. However, in contrast, some participants [24, 33] had significantly higher levels of fear of cancer reoccurrence compared to the fear of COVID-19. Other psychological disturbances reported included how the pandemic negatively affected their sleep [22, 23], which compounded a lack of energy and tiredness [24] with temper outbursts [22]. There was also a positive association between fear of COVID-19 and increased symptoms of psychological distress and unmet psychological needs [32, 34].

### Patient-clinician communication needs

Across the included studies, many expressed concerns with the transition to virtual appointments with their health care team, noting that the previous model of in-person appointments provided reassurance and feelings of comfort [35]. In contrast, telehealth appointments meant it was easier to just agree with their clinician and many patients forget to ask important questions that they wanted to ask:

I think because on the phone it’s very easy to just go, oh yeah everything, grand, and then the phone call is over in literally a minute or two and then you are like, oh I forgot to ask them that [31] page 8.

Many individuals affected by cancer during the COVID-19 pandemic expressed that the quality-of-care carried out over telehealth was suboptimal compared to in-person consultations. In contrast, only one participant positively noted that telehealth saved a lot of travel time in their day [35]. The transition to telehealth similarly restricted opportunities to engage with health care professionals, with some participants noting a change to empathy, and an attitude to blame COVID-19 for everything that goes wrong [31], while others displayed compounding uncertainty around the next steps in their treatment plans due to lack of interaction with their multidisciplinary care team [23]. Patients reported struggling with opting for support from health care professionals in what might be viewed as them being bothersome, but all they needed and wanted was just their normal follow-up for cancer care [35]. Lastly, some participants reported being most concerned with the reduction in hospital care and access to their care team because of the consequences of reduced face-to-face hospital appointments resulting from COVID-19 infection control policies [27].

### Health system and information needs

Many participants represented in the included studies reported additional anxiety and fear during the COVID-19 pandemic when required to attend hospital. Some highlighted concerns regarding the infection control measures and how they varied between patients and staff:

None of the patients were wearing masks and you know there was a lady in the bed, you know in the next bed to me who had a cough you know, not COVID but just, you know, things like that [31] page 7.

While others expressed:

I felt safe enough, they [hospital staff] were all wearing their masks and their gloves, and I found the whole place spotless as well. [31] page 7.

Some participants commented on the lack of available personal protective equipment (PPE) available to hospitals during different stages of the pandemic. Some participants noted that at the beginning of the pandemic accessing masks, gloves, and disinfectants came at high cost to them [30]. Participants often presented common concerns regarding interruptions to the quality and accessibility of care, flow and re-direction of service, and disturbances to the participation in clinical trials [19, 20, 25, 28]. Furthermore, scheduling issues, treatment delays, and miscommunication with health care teams were particularly concerning among the young adolescent cancer survivors [23]. Lastly, participants demonstrated confusion regarding government regulations and testing requirements when attending hospitals and noted that these did not align in every situation. Additionally, participants reported distress that the public were not respecting recommendations in the community to keep others safe given they were living with cancer [30].

### Spiritual needs

The spiritual needs of people with cancer were rarely discussed throughout the included studies. These findings represent a potential lack of holistic assessment given the restrictions enforced for funerals, restricted visitors during in-patient, and out-patient hospital visits for people with cancer; however, some participants indicated feelings of peace over distress when describing end of life experiences in relation to COVID-19 in palliative patients [19]. People affected by brain cancer [25] expressed concerns with “end of life” care.

### Daily living and practical needs

People affected by head and neck cancers noted periods of social isolation, which were exacerbated at holiday times, when they could not have family or friends over to visit due to the increasing infection risk. Many individuals did not have a support system available to them, resulting in poor diets and reduced oral intake [29]. Findings from several studies observed that participants commented on problems with accessibility of food delivery services and food during shortages causing similar nutritional deficits [21]. Patients sacrificed their self-care due to work demands [36]. Few participated in exercise or health self-management behaviours. Practically, participants expressed varying issues with transport [20, 25], resulting in deficits in the accessibility of care [20, 32] or medications [21], and causing an overall disruption to their daily lives [22]. Many experienced financial toxicities due to reduced household income. Many participants expressed concerns about reduced household income from not being able to attend work [36] due to enforced lockdowns. Others alluded to issues with medical insurance or reimbursement [25, 26], loss of employment, financial difficulties, reduction to working hours, or being forced to take sick or take annual leave [23, 28]. Many participants reported distressed about not being able to engage in usual activities of living with a loss of work as an additional practical concern for them [24, 26]. Financial concerns were a significant predictor of psychological distress [34] with a positive association between loss of income and unmet needs.

### Interpersonal/intimacy needs

Only one study explored interpersonal/intimacy-related needs. Women with breast cancer were the only sample group that reported needs in relation to intimate relationships during the COVID-19 pandemic, noting statistically significant reductions in changes to sexual feelings, sexual relationships, and a lack of information regarding intimate relationships from their health care team during the pandemic [27].

### Family-related needs

Children and family represented a small portion of the participants included in this review. Survivors of childhood brain cancer and their caregivers [22] noted that the closure of schools and day-care facilities plus the family’s inability to care was disruptive to the family dynamic [25]. A positive association was reported between unmet needs and reduced support within the family to mobilise their own support [27]. Participants expressed feelings of isolation because they were not allowed to attend or support the patient during treatment or be present during discussions with the medical team due to COVID-19 restrictions [19, 23, 27]. The lack of family support during consultations leads to a significant change in opportunities that contributed to decisional regret in cancer treatment and care decision-making [27]. People affected by cancer also shared worries in relation to job security and the disruption that this causes to daily functioning and broader family-related needs [22, 23].

### Social needs

Socially, participants of the included studies collectively presented a common theme due to recurrent restrictions and lockdowns leading to feelings of isolation. Across the included studies, many sub-themes of isolation were expressed by participants. One participant noted that not allowing anyone into their home held a substantial impact on their family life as their usual support systems were not readily available to them. Illustrated by the following quote:

So you’re very much on your own all of the time, you suffer in silence because you know it will end [31] page 7.

Feelings of social isolation extend further beyond support within their home, to simple daily living needs [29] or support when attending hospitals for cancer treatment. People diagnosed with cancer needed to have their family or support network at their side as an important psychosocial aspect of care; however, during the pandemic, this was not allowed. Participants demonstrated they felt mostly isolated by visitor restrictions in hospitals during their treatment journey [25]. Additionally, people affected by cancer were forced to attend review appointments alone:

Let's say you're having a PET scan done to see if your if your cancer is gone and you don't have anybody there to celebrate with when you get a good report and you don't have anybody to cry with when you get a bad report. I just I can't imagine doing that all myself. [29] page 5.

Whereas others described having support during these appointments as peace of mind in knowing someone else can confirm what you have been told:

It’s so good to have that extra pair of ears to, you know, come home and discuss it with your partner or spouse and say this is what I heard, and they’ll say, ‘no, no, no, that’s not what . . . she said at all [35] page 4.

When people affected by cancer attended appointments alone, they were distressed and conveyed that they needed to have a support person:

I’d nobody with me, so nobody heard [the diagnosis], you know my husband wasn’t there, so I phoned him when I was outside and I was bawling on the phone, sure he thought I was going to die [31] page 8.

Participants with endocrine-based cancers reported isolation negatively affected their quality of life [26] and others shared feelings of loneliness due to lack of social interaction during treatment [31]. Patients alluded to a positive association between feelings of restriction due to the isolation and psychological distress which significantly impacted their mental well-being [20, 23, 32]. Some expressed that they felt less connected with family and friends due to restrictions and the stay-at-home orders imposed on all during the pandemic [22]. However, some individuals reported positive feelings toward the pandemic, with participants noting they had increased family time and a new perspective on life [31], positively commenting on the infection control measures in hospital making them feel safe. Some participants [23] reported that they felt a sense of resilience because of the pandemic and they could apply the coping strategies which they developed when they were diagnosed with cancer. Females with breast cancer positively commented on the additional support when coping with house based caring pressures due to the lockdowns and regulations [27].

## Discussion

This systematic review sets out to understand the supportive care needs of people affected by cancer during the COVID-19 pandemic. The COVID-19 pandemic has had a significant negative impact on humanity, but more ominously for those living with or affected by cancer. Due to their immunocompromised state, they are required to be hypervigilant toward social endeavours and increased infection control measures when accessing health care facilities whether in- or out-patient. This review critically examined evidence published between December 2019 and February 2022 to identify the experiences of supportive care needs among those living with cancer. It was evident that people living with cancer reported physical, psychological/emotional, cognitive, patient-clinician communication, health system/information, spiritual, daily living, interpersonal/intimacy, practical, family, and social needs specific to the impact of COVID-19. It was evident that patients reported reduced assess and availability for symptom management support during the pandemic, further compounded by the rapid implementation of telehealth services in cancer. These findings have important implications because many individuals experienced poor symptom management because of reduced access to health care services (particularly with problems with pain, nutrition, fatigue, and psychological well-being).

Service reconfiguration, accompanied by social distancing, lockdowns, and curfews, was evident to have a negative impact on people affected by cancer. While it was a necessity that patients needed to be managed, for the most part, without visits to hospitals to minimise infection risk, this was identified as a health care system concern. A significant global push and rapid adoption to transition to cancer care to telehealth services remains [3] but for many participants represented in this review they expressed that they had concerns with this model of care. Patients valued the face-to-face in-person consultations with their care team and found that often the telehealth model lacked empathy and compassion at times, with missed opportunities to ask questions and gain the information that they needed. While momentum continues for the sustained use of cancer telehealth services post-pandemic [37], researchers and health care professionals cannot disregard the important concerns expressed by patients during COVID-19. Further research is needed to identify appropriate risk stratified telehealth models of cancer care [3], to ensure that the challenges of connectivity, communication, and access for remote areas, including safeguarding the elderly and vulnerable patients, are fully addressed.

### Limitations

This systematic review has many strengths including the clear and specific methodology which followed a registered priori protocol. In addition, to the independent reviewer’s contributions throughout the entirety of the systematic review process, the study provided insights across heterogenous study populations in terms experiences of unmet supportive care needs experienced during COVID-19. There are several limitations worthy of comment. This integrative review only included peer-reviewed studies published in the English language and as such it may have limited our understanding of the wider global impact of the COVID-19 pandemic on those living with cancer with cultural differences. The studies included for review were also conducted in mostly developed and western nations potentially biasing the management and impact the COVID-19 pandemic had on its participants. Despite these limitations, the review team followed a transparent prior review methodology to improve the rigour and validity of the findings.

## Conclusion and implications for cancer survivors

The global negative consequences of the COVID-19 pandemic on experiences of supportive care for people living with cancer are evident. This review has identified important areas of unmet supportive care needs, which require careful consideration in the future development of cancer care services. The results of this review may also be applied to and used to inform the management of any future pandemics within cancer supportive care. Although the current emphasis is on managing COVID-19, the focus soon must centre on the recovery plan and restoration of the balance of cancer care in the era of COVID-19 and beyond.

## Supplementary Information

Below is the link to the electronic supplementary material.

Supplementary file1 (pdf 15.3 KB)

Supplementary file2 (pdf 47 KB)

Supplementary file3 (pdf 24 KB)

## References

[1] World Health Organisation. Cancer WHO. 2022. Available from: https://www.who.int/news-room/fact-sheets/detail/cancer. Accessed June 2022.

[2] World Health Organisation. WHO Director-General’s opening remarks at the media briefing on COVID-19 - 11 March 2020 WHO: WHO; 2020. Available from: https://www.who.int/director-general/speeches/detail/who-director-general-s-opening-remarks-at-the-media-briefing-on-covid-19---11-march-2020. Accessed June 2022.

[3] Paterson C, Bacon R, Dwyer R, Morrison KS, Toohey K, O’Dea A, et al. The role of telehealth during the COVID-19 pandemic across the interdisciplinary cancer team: implications for practice. Seminars in Oncology Nursing. 2020;36(6):151090. 10.1016/j.soncn.2020.151090 PMC7561334 33218886

[4] Paterson C, Gobel B, Gosselin T, Haylock PJ, Papadopoulou C, Slusser K, et al. Oncology nursing during a pandemic: critical reflections in the context of COVID-19. Semin Oncol Nurs. 2020;36(3):151028. 10.1016/j.soncn.2020.151028 PMC7177078 32423833

[5] Sutherland K, Chessman J, Zhao J, Sara G, Shetty A, Smith S (2020). Impact of COVID-19 on healthcare activity in NSW, Australia. Public Health Research Practice.

[6] O'Dea A, Gedye C, Jago B, Paterson C. Identifying the unmet supportive care needs of people affected by kidney cancer: a systematic review. J Cancer Surviv. 2021. 10.1007/s11764-021-01113-8. 10.1007/s11764-021-01113-8 34595697

[7] Paterson C, Robertson A, Smith A, Nabi G (2015). Identifying the unmet supportive care needs of men living with and beyond prostate cancer: a systematic review. Eur J Oncol Nurs.

[8] Paterson C, Primeau C, Bowkerm M, Jensen B, MacLennan S, Yuan C, et al. What are the unmet supportive care needs of men and their partner/caregivers living with and beyond penile cancer? 2018;48:101805. 10.1016/j.ejon.2020.101805. 10.1016/j.ejon.2020.101805 32947156

[9] Paterson C, Jensen B, Jensen J, Nabi G (2018). Unmet informational and supportive care needs of patients with muscle invasive bladder cancer: a systematic review of the evidence. Eur J Oncol Nurs.

[10] Paterson C, Kozlovskaia M, Turner M, Strickland K, Roberts C, Ogilvie R (2021). Identifying the supportive care needs of men and women affected by chemotherapy-induced alopecia? A systematic review. J Cancer Surviv.

[11] Doyle R, Craft P, Turner M, Paterson C. Identifying the unmet supportive care needs of individuals affected by testicular cancer: a systematic review. J Cancer Survivorship. 2022. 10.1007/s11764-022-01219-7 10.1007/s11764-022-01219-7 PMC10960773 35781623

[12] Hart NH, Crawford-Williams F, Crichton M, Yee J, Smith TJ, Koczwara B, Fitch MI, Crawford GB, Mukhopadhyay S, Mahony J, Cheah C, Townsend J, Cook O, Agar MR, Chan RJ (2022). Unmet supportive care needs of people with advanced cancer and their caregivers: a systematic scoping review. Crit Rev Oncol Hematol.

[13] Hanna TP, King WD, Thibodeau S, Jalink M, Paulin GA, Harvey-Jones E, et al. Mortality due to cancer treatment delay: systematic review and meta-analysis. bmj. 2020;4;371:m4087. 10.1136/bmj.m4087. 10.1136/bmj.m4087 PMC7610021 33148535

[14] Whittemore R, Knafl K (2005). The integrative review: updated methodology. J Adv Nurs.

[15] Page MJ, McKenzie JE, Bossuyt PM, Boutron I, Hoffmann TC, Mulrow CD (2021). The PRISMA 2020 statement: an updated guideline for reporting systematic reviews. Int J Surg.

[16] World Health Organisation. COVID-19 China WHO. WHO 2019. Available from: https://www.who.int/emergencies/disease-outbreak-news/item/2020-DON229. Accessed June 2022

[17] Boyes A, Girgis A, Lecathelinais C (2009). Brief assessment of adult cancer patients’ perceived needs: development and validation of the 34-item Supportive Care Needs Survey (SCNS-SF34). J Eval Clin Pract.

[18] Hong QN, Fàbregues S, Bartlett G, Boardman F, Cargo M, Dagenais P (2018). The Mixed Methods Appraisal Tool (MMAT) version 2018 for information professionals and researchers. Educ Inf.

[19] McFarlane P, Halley A, Kano Y, Wade N, Wilson S, Droney J (2022). End-of-life experiences for cancer patients dying in hospital with COVID-19. J Patient Experience.

[20] Büntzel J, Micke O, Klein M, Büntzel J, Walter S, Keinki C (2021). Take care or “German Angst”? Lessons from cancer care during COVID-19 pandemic in spring 2020. J Cancer Res Clin Oncol.

[21] Davis K, Wilbur K, Metzger S, Garcia SF, Cahue S, Webster K, et al. Symptom and needs assessment screening in oncology patients: alternate outreach methods during COVID-19. Philadelphia, Pennsylvania: Taylor & Francis Ltd; 2021. Report No.: 0734–7332. 10.1080/07347332.2021.1890663 33792515

[22] Fisher AP, Patronick J, Gerhardt CA, Radonovich K, Salloum R, Wade SL (2021). Impact of COVID-19 on adolescent and emerging adult brain tumor survivors and their parents. Pediatr Blood Cancer.

[23] Shay LA, Allicock M, Li A. “Every day is just kind of weighing my options.” Perspectives of young adult cancer survivors dealing with the uncertainty of the COVID-19 global pandemic. J Cancer Survivorship : Res Pract. 2021. 10.1007/s11764-021-01069-9 PMC8200324 34125379

[24] Yildiz Kabak V, Atasavun Uysal S, Duger T (2021). Screening supportive care needs, compliance with exercise program, quality of life, and anxiety level during the COVID-19 pandemic in individuals treated with hematopoietic stem cell transplantation. Support Care Cancer : Off J Multinatl Assoc Support Care Cancer.

[25] Amidei C, Arzbaecher J, Maher ME, Mungoshi C, Cashman R, Farrimond S, et al. The brain tumor not-for-profit and charity experience of COVID-19: reacting and adjusting to an unprecedented global pandemic in the 21st century. Neuro-oncol Adv. 2020;3(1):vdaa166. 10.1093/noajnl/vdaa166 PMC7798794 33501430

[26] Falcone R, Grani G, Ramundo V, Melcarne R, Giacomelli L, Filetti S (2020). Cancer care during COVID-19 era: the quality of life of patients with thyroid malignancies. Front Oncol.

[27] Hulbert-Williams NJ, Leslie M, Hulbert-Williams L, Smith E, Howells L, Pinato DJ (2021). Evaluating the impact of COVID-19 on supportive care needs, psychological distress and quality of life in UK cancer survivors and their support network. Eur J Cancer Care.

[28] Osterman CK, Triglianos T, Winzelberg GS, Nichols AD, Rodriguez-O’Donnell J, Bigelow SM, et al. Risk stratification and outreach to hematology/oncology patients during the COVID-19 pandemic. Support Care Cancer : Off J Multinatl Assoc Support Care Cancer. 2021;29(3):1161–4. 10.1007/s00520-020-05744-y PMC7549726 33047163

[29] Kirtane K, Geiss C, Arredondo B, Hoogland AI, Chung CH, Muzaffar J, et al. “I have cancer during COVID; that’s a special category”: a qualitative study of head and neck cancer patient and provider experiences during the COVID-19 pandemic. Support Care Cancer : Off J Multinatl Assoc Support Care Cancer. 2022. 10.1007/s00520-021-06773-x PMC8799415 35091844

[30] Mirlashari J, Ebrahimpour F, Salisu WJ (2021). War on two fronts: experience of children with cancer and their family during COVID-19 pandemic in Iran. J Pediatr Nurs.

[31] Drury A, Eicher M, Dowling M (2021). Experiences of cancer care during COVID-19: phase 1 results of a longitudinal qualitative study. Int J Nurs Stud Adv.

[32] Caston NE, Lawhon VM, Smith KL, Gallagher K, Angove R, Anderson E (2021). Examining the association among fear of COVID-19, psychological distress, and delays in cancer care. Cancer Med.

[33] Dimelow J, Lowe D, Rogers SN. Balancing patients’ fears of recurrence and fears of COVID-19 when considering their preference for review consultations. European archives of oto-rhino-laryngology : official journal of the European Federation of Oto-Rhino-Laryngological Societies (EUFOS) : affiliated with the German Society for Oto-Rhino-Laryngology - Head and Neck Surgery. 2021;278(11):4441–8. 10.1007/s00405-021-06662-3 PMC7882040 33582851

[34] Zomerdijk N, Jongenelis M, Short CE, Smith A, Turner J, Huntley K (2021). Prevalence and correlates of psychological distress, unmet supportive care needs, and fear of cancer recurrence among haematological cancer patients during the COVID-19 pandemic. Support Care Cancer : Off J Multinatl Assoc Support Care Cancer.

[35] Kilgour HM, Galica J, Oliffe JL, Haase KR (2021). The needs of older adult cancer survivors during COVID-19: implications for oncology nursing. Semin Oncol Nurs.

[36] Salha LA, Silva JCS, Martins CA, Araújo CSdC, da Silva EAS, Alves AG, et al. Caregivers of individuals with cancer in the COVID-19 pandemic: a phenomenological study. Int J Environ Res Public Health. 2021;19(1). 10.3390/ijerph19010185 PMC8751169 35010444

[37] Burbury K, Wong ZW, Yip D, Thomas H, Brooks P, Gilham L (2021). Telehealth in cancer care: during and beyond the COVID-19 pandemic. Intern Med J.

