# Supplementary material for: The unmet supportive care needs of people affected by cancer during the COVID-19 pandemic: an integrative review
Source: J Cancer Surviv. 2022 Oct 29;17(4):1036–56. doi: 10.1007/s11764-022-01275-z (PMC9616701; doi:10.1007/s11764-022-01275-z)
Supplement: Supplementary file 1 — Supplementary file1 (pdf 15.3 KB) [file 11764_2022_1275_MOESM1_ESM.docx]

**Supplementary Table 1.** Electronic database searched and search terms used.

| Database: Medline on EBSCOhost platform | | | |
| --- | --- | --- | --- |
| Date of search: 24/02/22 | | | |
| Symbols used in this document: | | | |
| “ ” - finds a phrase | | | |
| **Search #** | **Concept/Explanation** | **Search Terms/Strategy** | **# of Results** |
| **#1** | Unmet Supportive Care | “unmet supportive care needs” OR “unmet needs” OR “care needs” OR “patient needs” OR “needs assessment” OR “family needs” OR “caregiver needs” OR “supportive care needs” OR “person centred needs” OR “holistic needs” | 8,794 |
| #2 | Cancer | OR neoplasms | 513,303 |
| #3 | Cancer AND Unmet Supportive Care Needs | #3 and #4 | 242 |
| #4 | COVID-19 | OR coronavirus OR sars-cov-2 OR cov-19 OR 2019-ncov | 227,165 |
| #5 | Unmet supportive care AND Cancer AND Covid-19 | #1 AND #2 AND #4 | 84 |
| Limiters | Date | December 2019- February 2022 |  |

| Database: CINAHL Plus with Full text on EBSCOhost platform | | | |
| --- | --- | --- | --- |
| Date of search: 24/02/22 | | | |
| Symbols used in this document: | | | |
| “ ” - finds a phrase | | | |
| **Search #** | **Concept/Explanation** | **Search Terms/Strategy** | **# of Results** |
| **#1** | Unmet Supportive Care | “unmet supportive care needs” OR “unmet needs” OR “care needs” OR “patient needs” OR “needs assessment” OR “family needs” OR “caregiver needs” OR “supportive care needs” OR “person centred needs” OR “holistic needs” | 7,245 |
| #2 | Cancer | OR neoplasms | 116,111 |
| #3 | Cancer AND Unmet Supportive Care Needs | #3 and #4 | 834 |
| #4 | COVID-19 | OR coronavirus OR sars-cov-2 OR cov-19 OR 2019-ncov | 89,003 |
| #5 | Unmet supportive care AND Cancer AND Covid-19 | #1 AND #2 AND #4 | 30 |
| Limiters | Date | December 2019- February 2022 |  |

| Database: APA PSYC Info on EBSCOhost platform | | | |
| --- | --- | --- | --- |
| Date of search: 24/02/22 | | | |
| Symbols used in this document: | | | |
| “ ” - finds a phrase | | | |
| **Search #** | **Concept/Explanation** | **Search Terms/Strategy** | **# of Results** |
| **#1** | Unmet Supportive Care | “unmet supportive care needs” OR “unmet needs” OR “care needs” OR “patient needs” OR “needs assessment” OR “family needs” OR “caregiver needs” OR “supportive care needs” OR “person centred needs” OR “holistic needs” | 1,926 |
| #2 | Cancer | OR neoplasms | 9,238 |
| #3 | Cancer AND Unmet Supportive Care Needs | #3 and #4 | 228 |
| #4 | COVID-19 | OR coronavirus OR sars-cov-2 OR cov-19 OR 2019-ncov | 14,342 |
| #5 | Unmet supportive care AND Cancer AND Covid-19 | #1 AND #2 AND #4 | 7 |
| Limiters | Date | December 2019- February 2022 |  |
