# Supplementary material for: The unmet supportive care needs of people affected by cancer during the COVID-19 pandemic: an integrative review
Source: J Cancer Surviv. 2022 Oct 29;17(4):1036–56. doi: 10.1007/s11764-022-01275-z (PMC9616701; doi:10.1007/s11764-022-01275-z)
Supplement: Supplementary file 2 — Supplementary file2 (pdf 47 KB) [file 11764_2022_1275_MOESM2_ESM.docx]

**Supplementary Table 2** Qualitative Findings

| **Authors:** Drury A et al., 2021 |  |  | | | | |  |
| --- | --- | --- | --- | --- | --- | --- | --- |
| **Findings (themes in papers)** | **Illustrations (Page number)** | | **Evidence** | | | **Finding number** | |
|  |  |  | **Unequivocal** | **Credible** | **Unsupported** |  | |
| 1. Being careful, keeping safe and feeling safe   Recognised their increased risk of infection and endeavoured to minimise their risks of contracting COVID-19 through shielding and cocooning measures. Avoiding public places even for essential purposed such as shopping. | *I was very careful I mean I didn’t go into a shop from March until ... probably October or November even (7)*  *I was terrified. I was terrified. No other way of describing it, [laughing] our shopping would be delivered, and I hated shopping day because we had to clean every item down before it came in. (7)*  *[...] they [children] wanted to go out and play with the kids out the back, as time went on it was hard to keep them in. We were trying our best to keep them away from other people. We were told you know that was important. But that was the biggest challenge as opposed to anything else. (7)*  *There is a massive risk coming into our house every day but we’re very lucky, we’re clean, we have to extra sanitize and to do 3 h cleaning extra that I never did before. And sometimes you just, you have no choice. I can’t bring anyone in, I have no one to help me, I have no support, my family don’t live near me. And I can’t risk a friend of mine coming in case she’s been around someone. So, you’re very much on your own all of the time, you suffer in silence because you know it will end. (7)* | | **X** |  |  | **F1** | |
| 1. Being careful, keeping safe and feeling safe   (II) Despite implementing shielding measures, for some participants, admission to hospital or attendance at hospital appointments was unavoidable. Several participants described anxiety and distress at the prospect of attending these appointments and being admitted to hospital. | *I probably was a bit more concerned when I was in hospital after the surgery as well, just you know, even within the ward. None of the patients were wearing masks and you know there was a lady in the bed, you know in the next bed to me who had a cough you know, not COVID but just, you know, things like that. I think you know, would make you a bit more on edge, which if it was another year you probably wouldn’t think anything of it. But again, you know it’s only smaller things like that. (7)* | | **X** |  |  | **F2** | |
| 1. Being careful, keeping safe and feeling safe   (III) Those who attended hospital appointments spoke positively of safety measures taken by hospitals to protect patients including pre-appointment COVID questionnaires, use of PPE and visibility for cleaning and disinfection in public areas. | *I felt safe enough, they [hospital staff] were all wearing their masks and their gloves, and I found the whole place spotless as well. I had no trouble that way, no worries at all [...] it’s [COVID] had made people more hygienic you know what I mean, more cleaning I suppose compared to maybe when I thought about hospitals before they are more sterilized now. (7)*  *If anything, I’d probably nearly feel more nervous of other patients, or you know, sometimes some people can be a bit more lax you know. I suppose with how they wear their masks. [...] I’ve seen the difference since I initially went to the hospital and went to the various clinics; just you know, it was unrecognizable. I suppose just the amount of people who were in the clinics, you know, at that stage compared to what they were later in the year. (7)*  *I did feel very safe like, all the social distancing, the nurses would ring you like regarding COVID before you went in and like when you got there you were tested and brought in different doors. You felt very, very safe. So then it was just a matter of getting your chemo and getting home. (7)*  *[...] you feel that you are prioritized more and it’s a more organized system in place perhaps to get things moving through. (7)*  *He was in hospital twice and each time was for about a week, it really took its toll mentally on him. Because again, the symptoms he had, he had to be isolated. And you know, simple things like being able to work the television, you know and he didn’t have, you know just the company or the chitchat or whatever. (7)* | | **X** |  |  | **F3** | |
| 1. Shrinking supports and feeling isolated   (I) The experience of being alone for hospital visits at critical points in their diagnosis, treatment and follow-up care was described by several participants. While participants noted efficiencies in cancer services, several described the isolation, loneliness and distress of attending hospital appointments without their support networks. | *I’d nobody with me, so nobody heard [the diagnosis], you know my husband wasn’t there so I phoned him when I was outside and I was bawling on the phone, sure he thought I was going to die [...] (8)*  *Then with radiotherapy you’d have to, you’d arrive, you’d have to ring and wait outside and be called in have your temperate taken. Go back to the car and wait in the car until you were called to go into radiotherapy. And then you wouldn’t meet anyone until you went in, you met your radiation therapist. They were cleaning down when you went in which was comforting, but I had to go on my own. Nobody could travel with me because we were in, it was in lockdown. And so, I found it lonely in the car on my own. I found my first session really, really stressful. I was crying coming home and I was never going back again. I found, I hated it, I hated every minute of my radiotherapy. (8)* | | **X** |  |  | **F4** | |
| 1. Shrinking supports and feeling isolated   (II) However, many narratives highlighted how COVID-19 dominated the cancer care agenda during appointments and when seeking advice, restricting opportunities for engagement with healthcare professionals and the person-centredness of care. Some believed that healthcare professionals’ preoccupation with COVID- 19 compromised empathy and care, acting as a barrier to care and support during treatment. | *[...] in the last year and a half when I was first diagnosed, I noticed the empathy has changed completely since COVID. There’s a lack of empathy. There is very much a lack of communication from the hospital to the patients. And really without sounding very dramatic, there’s a lack of care at the moment because they seem to presume that COVID only exists now, even in the hospitals its very much well COVID, well COVID, well COVID [...] it’s like they don’t want to communicate, they don’t feel they need to now, because COVID has taken over from their care, so they can use any excuse they want to now and they can blame it on COVID. (8)*  *Trying to ring and just get information about how he was, it was very difficult. And even there was one instance where he rang me, and he was sitting in a chair for hours because there were no beds [...] or he’d ring me because he was freezing cold in a place where he was, and I’d have to try and ring to see was there anyone that could give him a blanket. So there was those two bits, I suppose, out of the whole year, that you know it just, it was very frustrating. But again, you know, look it I understand, I know myself the way things work. (8)* | | **X** |  |  | **F5** | |
| 1. Shrinking supports and feeling isolated   (III) The transition to telephone consultations for some participants was an added barrier to support and limited person-centeredness in care interactions. | *I just feel at the moment that I’m not being listened to, you know, one of my phone consultations back during COVID lasted 2.5 min. (8)*  *[...] I suppose it’s nicer to sit there with the consultant because I suppose you have more of an opportunity to, to you know I suppose go into things with them you know if you need to. The face-to-face I suppose is a bit more personal always isn’t it. So, you know I had missed that a little bit but look, I understand the way things are, and you know it’s for our own safety, and they don’t want to be bringing people in needlessly like, you know. (8)*  *[...] it’s not ideal I guess in a way you kind of need to be maybe a bit more prepared, I think because on the phone it’s very easy to just go, oh yeah everything, grand, and then the phone call is over in literally a minute or two and then you are like, oh I forgot to ask them that, or I didn’t bring that up or whatever. I think you know when you are face-to-face, it’s more of a natural conversation, it’s more, you know, you are more inclined to maybe talk about things maybe a little bit more. (8)*  *COVID hasn’t reached my hospital yet, but yet any time I enter the hospital or speak to a nurse, they’re very much like, they bring in the COVID line, which doesn’t, it doesn’t wash with me as a patient. They try to insult your intelligence by thinking that this will take back, you know, to times where you’d ring with a sore pain, or you’d ring with the side effects that they want you to ring with. But now, you don’t get a call back for two days, even though COVID is not in their hospital. That’s what I see a lot of. (8)*  *I actually called them up [the cancer care team] for the first time in a long time about the vaccines because I was a bit concerned about any interaction between Tamoxifen and the vaccines, and you know, between that and the fake news that you hear like I just wanted more advice. And so, I left a voicemail, and within two days, she got back to me; we had a long conversation. So, no, I do feel, you know, I feel quite well supported by them. (8)* | | **X** |  |  | **F6** | |
| 1. Shrinking supports and feeling isolated   (IV) Several participants also acknowledged how the variety of peer support and cancer support services that would be available under normal circumstances were no longer accessible to them. | *[...] when it [radiotherapy] started, I found it very lonely because I was used to in chemo, my mother would come with me to all my chemo sessions. And there would be in [hospital] there’s four patients sit around a table, and their family members can sit with them, we used to have a great laugh at chemo. [...] (8)*  *[...] you actually don’t get the chance to chat to anybody. That’s the only, I suppose, negative side of COVID; you don’t get anyone to talk to through the whole thing. (8)*  *I suppose the one thing that I found difficult with it all, because of COVID, it was difficult to reach out for support from a mental health point of view, because normally you would have [cancer support centre]. And they shut down kind of petty much completely other than you know phone support. But they offer a whole range of other services, and I wasn’t able to avail of any of those and they also have services for children and partners as well. None of that was available. (9)*  *We did as much as we could ... I’d leave; you know, medicines, or whatever, at the door, and she would kind of, she would be up at night with him you know when he was sick. (9)* | | **X** |  |  | **F7** | |
| 1. Not missing out   Many felt that COVID-19 had introduced a new-found perspective on life, whereby the appreciated the opportunity to foster closeness with their families. | *It’s just being close to them, so the positive I can take is just, kind of, figuring out what’s really important. And its family and just being able to kind of spend time with them while I can. It’s been cruel. Just the distance and looking in the window when someone is so sick and not being able to, the helplessness not being able to do anything about it for fear. (9 )*  *I suppose everybody is in the same boat. I feel I’m not missing out as much. (9)*  *[...] I’ve been saved because of COVID, from walking down the streets and having 20 million people looking at my baldy head and my no eyebrows; and ask me the question, and have to deal with the ’oh I’m so very sorry to hear that,’ etc., etc., etc. Which I can imagine, at the start, when you’re told you have cancer, every time that you have to tell someone else, it’s like hearing the news all over again. So, I think in that sense, because of COVID, I was saved a lot of that. I was able to do a lot of it on my own terms. I was able to manage the flow of information. (9)*  *Everybody keeps looking at me when, you know, when I was completely bald, and I was thinking, well I don’t have to go anywhere so nobody else seen me [laugh]. (9)* | | **X** |  |  | **F8** | |

| **Authors:** Kilgour. H et al., 2021 |  |  | | | | |
| --- | --- | --- | --- | --- | --- | --- |
| **Findings (themes in papers)** | **Illustrations (Page number)** | | **Evidence** | | | **Finding number** |
|  |  |  | **Unequivocal** | **Credible** | **Unsupported** |  |
| 1. Concerns about virtual versus in person appointments | *“It’s just reassuring to see them in person, to see their face, to watch their non-verbal. You know what I mean? It’s just nicer.” (3)*  *“There [was] something wrong and she [oncologist] knew right away that there was a problem. But had that been a phone-call, I don’t think she’d have ever diagnosed it.” (3)*  *“I’ve had three appointments in the pandemic, and I haven’t spoken to my actual staff person yet.” (3)*  *“Well, all my appointments were across the phone. . . .I don’t think they’re as good as the in-person ones. I guess it saved us a lot of trips because . . . well, if I go to appointments, half a day is shot by the time we drive there and back, . . . but I would sooner have the in-person appointments when you’re only having them every six months or so anyways.” (3)* | | **X** |  |  | **F9** |
| 1. Barriers to Caregiver Attendance at Appointments | *“Well, the main difference, the main problem is the fact that she can’t come in with me,” explaining, “we do everything together.” (3)*  *“It’s so good to have that extra pair of ears to, you know, come home and discuss it with your partner or spouse and say this is what I heard, and they’ll say, ‘no, no, no, that’s not what . . . she said at all.’ . . . So, I think it’s really important that when people get back on track that they are allowed to take a person with them to appointments, because it’s so, so beneficial.” (4)*  *“I’m finding that my memory is poorer, and I don’t always think of things. So, with [partner’s name] comments, they’re very pertinent.” (4)*  *One participant described how they had initially attempted to record their appointments with their oncologist, however, noted this was challenging and that they “haven’t been doing that lately.” (4)*  *“what I started to do was I made a list of questions myself. So, when I went in . . . I didn’t forget any questions I would jot down the answers. So, when I couldn’t take anybody that was fine.” (4)* | | **X** |  |  | **F10** |
| 1. Impediments to Accessing Health Care Services | *“It’s a little disappointing because the COVID-19 has really backed up any type of appointment you can get. And you know it’s gone from a couple weeks to three or four months for things.” (4)*  *“Have been put on hold since the pandemic,” (4)*  *“They want you to do these follow-up check-ups, but you can’t get in [to see them].” (4)*  *“with the systems and the pressure on the system right now, I want to do everything I can to stay as healthy as I can. Mentally and physically.” (4)*  *“The whole social structure is under pressure, so the less you have to rely on other people or other services the better off you’ll be, I think.” (4)*  *“You don’t really want to be bothersome with what’s relatively minor compared to what they’re really dealing with.” (4)*  *“I don’t think it had anything to do with the pan- demic. To me it’s irrelevant. You know? Just a fear that you have of the results—and no matter what’s going on in the world.” (4)*  *“It’s lasting longer, and I always think if the cancer’s going to come back it’s probably not going to be the first six months, right? It’ll be you know, maybe a year, year into it you know, and so maybe as it gets longer—or if COVID goes longer I may have more concerns about cancer recurrence and the support. (4)*  *If there is a complication or say the cancer resurfaces, will treatment and access to facilities be available? And we all know the pressure that’s being put on hospitals and ICUs and surgeries. And they mentioned cancer surgeries that are being put on the back burner or cancelled until further notice. So, if it comes up again, it might be a lot different than the first time.” (4)* | | **X** |  |  | **F11** |

| **Authors:** Kirtane. K et al., 2021 |  |  | | | | |  |
| --- | --- | --- | --- | --- | --- | --- | --- |
| **Findings (themes in papers)** | **Illustrations (Page number)** | | **Evidence** | | | **Finding number** | |
|  |  |  | **Unequivocal** | **Credible** | **Unsupported** |  | |
| 1. Increased Psychological distress | *It contributes to a little bit of anxiety because, you hear over and over and over, people that are in a higher risk category. And, you know, by you being diagnosed with cancer, you’re definitely put to the top of that list. So, it’s very worrisome. ‘Are you going to get COVID while you’re getting treatment? ...What is that gonna do to your body? Are you even going to be able to deal with that? Are there medicines that are going to help you through that?’ Just a lot of questions in your mind. (4)*  *“COVID is stressful to everybody right now... I think that we're worried about lots of stuff and, you know, throw the cancer in on top of it, it's like a double whammy.” (5)*  *“I'm more careful when I do go out. I wear a mask...I always consider myself clean, but I take it to another level; I come in and I take a shower for just going out to a grocery store. I don't know if it’s a phobia...I guess that's the best way I can describe it.” (5)*  *“I think a lot of [patients] are just very anxious... because they're receiving chemotherapy and they're potentially immunocompromised.” (5)* | | **X** |  |  | **F12** | |
| 1. Exacerbated social isolation | *One of the less obvious symptoms would be social isolation...let’s just say it’s Thanksgiving time...they’re not going to be able to eat typical foods with other people...so that adds a social component to this as well, unfortunately. (4)*  *Because of COVID, I was by myself over there... if I would have had that support, I may have eaten better; for someone to be there to push me to drink a Boost or you’re going to make [food] for me. Because I had to get up and make it myself, which was tiresome in and of itself. But, you know, I got to say a few times I said, ‘Gee, I wish my mother would fix me breakfast’ or something like that... and you’d sit there in bed and think, ‘I got to get up and eat. I got to get up and eat.’ But, you know, an hour later, you still find yourself sitting in bed and you still haven’t eaten. (4)*  *I would say the that the pandemic has had at least as much to do with its effect on my personal relationships as my cancer treatment has because I was told very early on to pretty much just sequester myself because of the radiation and chemotherapy treatment. Any exposure to COVID-19 would be extraordinarily difficult for me to survive... So, it's had an obviously large effect on all my relationships.” (5)*  *“I was scared my resistance [to COVID-19] was low, so I didn't go anywhere... I didn't even go to the grocery store because I was worried about getting sicker. So, for a couple of months, I don't want to say I didn't have a life, but I didn't do anything but what I had to around the house. That was it.” (P5)*  *“I also have another illness that I've had for 33 years, which is an immunity disease. My immune system is very low, so having cancer along with that, we've been isolating ourselves since March, my wife and I. And so we haven't seen anybody except a doctor, the grocery store, or gas station for 8 to 7 months now, I guess. It's been very, very confined. We do have social things on Zoom and other medias that we frequent. But of course, it's not the same as being with someone physically.” (5)*  *“This just leads to isolation, that’s all. And again, if this is a different time frame with no COVID, it would be different...Number one, I didn’t want anybody coming over. Number two, I can’t eat, you know? They’re like, ‘Well, what do you like to eat? We’ll make you food.’ I said, ‘You’re great and I love you guys, but you’re not understanding. I can’t even swallow liquid right now. That’s how bad the sores are.’ So, I think they begin to understand that. So, it's just the isolation. And again, I think I'm an outlier because it is, I have cancer during COVID. So that's...kind of a special category. (5)* | | **X** |  |  | **F13** | |
| 1. Added stress in clinic for patients and providers | *I really don't like the fact that when I go to a hospital, even Moffitt, my wife can't come in now. You know, she was with me through the whole thing...She's my sounding board when I'm asking questions. So that made it harder.” (5)*  *“Some [patients] are angry because Moffitt won't let their loved one in... What they're thinking about is what's going on in their world right now and they want that person. It's important for that person to be there with them and they can't... Let's say you're having a PET scan done to see if your if your cancer is gone and you don't have anybody there to celebrate with when you get a good report and you don't have anybody to cry with when you get a bad report. I just I can't imagine doing that all myself.” (5)*  *“I think one of the hardest things is when we could not have visitors come to our outpatient appointments... That was tough on everybody. That was tough from a clinician standpoint, making sure that the patient thoroughly was educated and understood the information we were giving them. And I think it was very hard on the patients because they rely on that social support and their family to be there at their appointments.” (5)*  *Not having my husband at the appointments because he comes, well, he used to come to every appointment that I took at Moffitt. So, him not being there, especially on those days when I was kind of nervous. Just like, when I had to do my scans; my first scans three months ago. And then, just dealing with like, not so pleasant news, but you're by yourself. I think that's been the most challenging part. (5+6)*  *It adds an extra layer onto what is already a stressful thing for the patient... A lot of them feel a little bit more alone, and just kind of enhances what they're feeling and their anxieties. And then, they're not able to focus on what we're telling them, some of the education and things like that... I found that I've had to repeat myself a little bit more on education things, or what types of things that we're wanting patients to do, just because they don't have that extra set of ears or that support system... You're having to give a lot more support to the patients, which you don't mind, but it just kind of drains you a little bit more than you're probably used to. So, you kind of take it home with you a little bit more. (6)* | | **X** |  |  | **F14** | |
| .   1. Delays in health care | *What has ended up happening is that a lot of patients have put a lot of their cancer care sort of on the back burner and have waited a long time to seek cancer care because they were just very afraid of COVID. That's something that we've seen a lot lately.” (5)*  *“When I first heard COVID on the increase, I missed y'all’s doctor's appointment. I don't want to go nowhere. I didn't want to take the risk. And then, of course, I came over and saw you like a month later.” (5)*  *“Well, I'm due a colonoscopy this year and they said they weren't handing out any appointments until after COVID calmed down. So, I'm still waiting for that appointment.” (5)*  *I wanted to see if [the swelling] would go down on its own, because I do tend to get swollen glands when I’m fighting any kind of illness. So, it didn’t go down and then that’s when...COVID hit. And so, I talked to a couple of friends of mine that are doctors, actually, and they’re like, ‘You know, I wouldn’t do any elective procedure right now,’ because COVID was starting to get really bad. And they said, ‘You don’t want to go to hospital for anything elective.’ So, I kind of waited a little bit, but then it never went down. (6)*  *I really haven't been to my primary care. I did one... tele-video thing, but I haven't had my annual physical and I haven't been to my dentist in 2020. So, [COVID- 19] changed that. (6)* | | **X** |  |  | **F15** | |

| **Authors:**  Mirlashari J et al., 2020 |  |  | | | | |  |
| --- | --- | --- | --- | --- | --- | --- | --- |
| **Findings (themes in papers)** | **Illustrations (Page number)** | | **Evidence** | | | **Finding number** | |
|  |  |  | **Unequivocal** | **Credible** | **Unsupported** |  | |
| 1. Swinging on the path of fear to adaptation    1. Exposed to an unknown and enormous threat | *“I have gotten used to cancer, I mean I know about it. However, corona is strange and unknown to me. So I am afraid of it more, and I am worried about getting the coronavirus.”(3).*  *“I am afraid of the corona. I am afraid of getting infected because everyone who gets it will die.”(3).*  *“The immune system of my child is weakened because of cancer. My child is at an increased risk of COVID-19.”(3).*  *“At the beginning of the outbreak, we had trouble getting masks, gloves, and disinfectants because of the shortages and high costs. Because of the cancer treatment, my child needs these.” (3)* | | **X** |  |  | **F16** | |
| 1. Swinging on the path of fear to adaptation    1. Developing strategies to address corona phobia | “*I was constantly following the corona news to get more information.” (3)*  *.“I was washing my hands constantly until my skin became dry. Whenever I touched anything, I washed my hands immediately because I was afraid of getting infected.” (3)*  *“We were terrified and anxious, but when we all stayed at home, the chance of getting the infection was reduced, and our anxiety was minimized.” (3)*  *“Even talking about corona is scary. I do not talk about it, and I do not listen to the news to make myself less worried.” (3)*  *“We try to follow hygiene principles and teach our children how and when to use a mask or wash their hands.” (3)*  *“In coordination with the doctor, we made the treatment program more compact and changed it to outpatient treatment.” (3)*  *“It is stressful for me that children with cancer are at increased risk of Coronavirus infection. However, when praying to God, I feel calm.” (3)* | |  |  |  | **F17** | |
| 1. Left alone at emotional distances | “It has been about two months since my father avoided close contact with me so that I do not get corona. We cannot communicate with our relatives. *Corona has created a gap between our loved ones and us and reduced affections.” (3)*  *“My daughter is very dependent on her father. She misses him so much. Children are very bored here.” (3)*  *“Fathers are not allowed to enter the ward, which makes children and their fathers upset.”(M5). (3)*  *“Playroom is closed. Children are not allowed to go out, and they are not allowed to play with other children.”(M6). (3)*  *“Children are not allowed to go to the Playroom. No one comes here to amuse them. Mothers and children are not allowed to go to other rooms or to talk in the corridors. The feeling of empathy and happiness of mothers and children is gone. I feel bored and lonely.”(C2). (3)*  *“It is tough for us. Corona has driven us crazy. We have been hospitalized for twenty days. My daughter misses her father. She doesn't eat. We are imprisoned in this room like a cage. We do not even leave the room, unless when they want to inject the medicine in my daughter's spinal cord. No one is allowed to visit us.”(M8). (3)*  *“Since nurses and doctors are using masks and shields, the children and their mothers are not able to identify them. Wearing mask along with social distancing in the unit has led to severe communication problems and intensifies the feeling of loneliness and emotional distancing among the children and their families.”(M10). (3)*  *“One of the favourite activities that brought me beautiful joy was atten ing school. When schools were closed, I lost my friends.”(C4).(4)*  *“We had a plan to travel, but it was cancelled because of the corona. My daughter was attending music classes, and she was in contact with her friends. However, because of corona and the quarantine, everything ceased, and she feels bored and lonely. I feel her anger.” (F1) (4)*  *“We had a lot of misfortune, the corona was added! Before corona, at least I used to take my son to the park once a month, but now I can no longer do that. Staying at home and being alone has made him more restless and troubled.” (m7) (4)* | | **X** |  |  | **F18** | |
| 1. Care system confusion and decreases quality of care    1. Sacrificing children and family’s needs due to concentration on COVID-19 prevention. | *“To be allowed to enter the ward, the mother and child must take a Corona test, which is expensive.”(M5). (4)*  *“We did not allow fathers to visit their children, who caused many objections, from families and the children themselves.”(N1). (4)*  *“Initially, I was not allowed to enter the ward. They said my daughter is old enough and does not need a companion. I spoke with the supervisor, and she said that if you had a corona test, you could enter the ward, and she informed the head nurse.”(F1). (4)*  *“As before, there was no disinfectant in all rooms and inside the ward. If necessary, we should have asked the nurses to deliver us some disinfectant.”(M2). (4)* | | **X** |  |  | **F19** | |
| 3.Care system confusion and decreases of quality of care  b. Confusion about issuing and implementing instructions | *“Some people do not respect public health instructions in the community or the hospital. Some people do not fully follow the principles of personal hygiene and health advice. Some doctors were using full personal protective equipment at the hospital, but some others only wore masks.”(M1).(4)*  *“Many people emphasized that mothers and children should be tested for corona before hospitalization, but these rules were only for us children. Nurses and doctors or other non-cancer patients were not required to observe these rules.”(M2). (4)*  *“Mothers and children were not allowed to enter the cancer ward with their shoes and were re- quired to use special slippers provided by the hospital. This is for our children's health, but why do they not observe this rule themselves and enter the wards with high heels? They are making it hard on us so that we do not carry corona to the ward. We do not even have the right to choose the right slippers for ourselves and our children, and we have to wear whatever they give us.”(M2). (4)* | | **X** |  |  | **F20** | |
| 3.Care system confusion and decreased quality of care  c. Children and Family expectations from medical staff and health system in the context of the COVID-19 pandemic. | *“These disinfectants are vital during the COVID-19 pandemic. When the pandemic is over, they no longer have value for us. We need these sub- stances, and they should provide sufficient disinfectant for us. We are faced with different stresses. We do not want the medical staff to upset us and make the situation worse by creating such tensions.”(M2).(4)*  *“The Corona test is expensive, and we cannot afford it. The head nurse or head of the cancer department should consider this issue and negotiate with the hospital to make this test free.”(F1). (4)*  *“Nurses are more nervous than before and stay away from us. They do not spend enough time to answer our questions, and they just lead us to our rooms and tell us not to leave the room.”(M2). (4)*  *“Some children want to play with me or talk to me or even hug me, but I have to avoid them. Because I have less time, and I have to observe COVID-19 related protocols.”(N1). (4)* | | **X** |  |  | **F21** | |

| **Authors:**  Salha A L et al., 2021 |  |  | | | | |  |
| --- | --- | --- | --- | --- | --- | --- | --- |
| **Findings (themes in papers)** | **Illustrations (Page number)** | | **Evidence** | | | **Finding number** | |
|  |  |  | **Unequivocal** | **Credible** | **Unsupported** |  | |
| 1. Sacrificing Self-care due to work demands | *It’s impossible to do any activity because you can’t leave the house (C1) (4)*  *I do not practice specific physical activities, only those related to housework and those related to remote work. (C17) (4)*  *I work most of the time, I don’t practice much activity because I take care of my father. (C35) (4)*  *Not very active. Walking. Around the block. Almost every day (3 to 4 times a week). (C42) (4)* | | **x** |  |  | **F22** | |
| 1. Worrying about the future: suffering and Unemployment | *My concern is about getting sick and not being able to take care of my father. (C4) (5)*  *...how I can survive without income... (C13) (5)*  *. . . staying healthy and taking care of emotional and financial health. (C17) (5)*  *. . . with my mother’s health! Fear of her contracting the virus because her immunity is low due to her treatment! (C33) (5)*  *. . . always hoping to improve. Because we trust in God always to give us strength. (C5) (5)*  *. . . get worse. We get worse every day in this life. (C12) (5) I hope to improve with the end of my son’s chemotherapy. (C26) (5) Look, I have faith in God that my health will improve in Jesus’ name. (C28) (5)*  *. . . and the emotional one too, with faith in God, that they will soon find the vaccine for this virus. (C30) (5)* | | **X** |  |  | **F23** | |
| 1. Complying with Public Health Rules as Closely as Possible | *I need to come and go all the time, but I take care of myself. (C19) (5)*  *In part, we are very careful to use a mask, hand sanitizer, hygiene, but I go to hospitals a lot. (C26) (5)*  *I started following all the protocols, but I had to keep going to work, even knowing that the number of infected and dead in my own service was getting closer to me. (C31) (5)*  *I’m avoiding crowding, but social isolation is a little difficult to meet due to my service. (C33) (5)* | | **X** |  |  | **F24** | |
| 1. Positive actions and reclaiming feelings in the post-pandemic world | *Give a hug to my children who live far away from me, I miss them a lot. (C1) (5)*  *Carefully go back, due to low immunity, to having contact with my family. (C2) (5)*  *Embrace the people I love! Including my mother who, due to the disease, we are taking great care to avoid, I will hug her a lot. (C33) (5)*  *Thank God for having passed this challenge. (C40) (5)*  *Thank God for taking care of me and my family, for not having COVID, thank God. (C41) (5)* | | **X** |  |  | **F25** | |

| **Authors:**  Shay. L Aubree et al., 2021 |  |  | | | | |  |
| --- | --- | --- | --- | --- | --- | --- | --- |
| **Findings (themes in papers)** | **Illustrations (Page number)** | | **Evidence** | | | **Finding number** | |
|  |  |  | **Unequivocal** | **Credible** | **Unsupported** |  | |
| 1. AYA behavioural responses to the pandemic    1. Social Distancing    2. Impact of social distancing on AYA’s | *“not when it became mandated but like when it became recommended by health officials” (4)*  *“I try to go to the grocery store for [my parents] be- cause they’re older, and I feel like I’m in better health than they are.” (4)*  *“very serious about staying far away from people who aren’t wearing face masks.” (4)*  *“I’m definitely seen as the bad guy in my family, because I don’t agree to birthday parties, I won’t agree to taking my mask off, I won’t go into people’s houses, I won’t let my child go into people’s houses, I won’t let people visit.” (4)*  *“I’m still in treatment. I still have cancer. Like, they just wouldn't get it. And so, I had to cut some of those friends off, which was unfortunate.” (4)*  *“I had a really good friend that got married in May, and so I kind of had this internal conflict back and forth of, the wedding still happened, and I wanted to go, and so it was just...there’s been a lot of situations and social situations like that that I feel like I’ve been having a lot of internal battle with what is the right thing to do.” (4)*  *“So yeah, there has been job changes. And not only, like, my role changing, but hours lost, financial status, conversations just get weird.” (4)*  *“I was really glad when the order came down that all these places you have to wear a mask every time you go in there. Because I just felt a sense of kind of like peace, that even though there are people who fight that, that it’s a we’re all in it together type of thing.” (4)*  *“So the cool thing, and I can’t believe I’m actually saying it's a cool thing, about COVID is that Stupid Cancer had their CancerCon virtually this year. And it’s something I probably would not have gone to in person, but I went to the digital CancerCon, and that's how I met [name redacted] And now I’m in an Instagram group chat with like a dozen other adolescent and young adult cancer survivors. I have my tribe. This is not something I had before this. You know, I’m almost four years out. Had not met a group of people, despite trying different things. This, it just wouldn't have happened without COVID and without having done the virtual confer ence.” (4)* | | **X** |  |  | **F26** | |
| 1. The added burden due to cancer    1. Difficulties and delays in medical care | *“I don’t care where on the totem pole you think I am, but I think I’m important enough to get that treatment.” (5)*  *“nothing’s changed” (5)*  *“I think I’m at the point where I go in every other year and don’t have to go in until next-next year.” (5)*  *“I really wanted to get the scans done. So, it was a little hard to have it delayed, but it’s just something I have to deal with.” (5)*  *But it’s not as easy as it was prior to COVID, right? Where you could call and say, “I need my MRI.” “Okay, what date are you available?” They’re asking me. Now I’m asking them, “What do you have avail- able?” And let me throw my whole schedule around just so I can make this appointment. Because I know if I don’t go to this appointment this week, I have to wait six weeks from now.” (5)*  *“It feels like a lot of the onus is on the patient. Instead of providing actual medical care, they’re just kind of giving you whatever you think you have is kind of what it feels like.” (6)*  *I think that, at least what I’ve experienced at the hospitals, like, I know that they are stressed, and they are losing out on all this money. And so it seems kind of like they're scheduling things for people by de- fault. And when you even bring up, like, is it safe, like, I'm worried about it, they’re...they admit that it’s not really safe and that you should probably just reschedule. But they’re scheduling everybody even though it’s not safe necessarily. And so that’s really not reassuring.” (6)*  *“felt like they [healthcare providers] weren’t taking it [COVID-19] as seriously as they should” (6)*  *“I’ve exhausted most of my baseline courses of treatment, so clinical trials are essential for me. And when you hear, like, oh, there’s one where you are an ideal fit, you fit all of the inclusion criteria, there’s no exclusion bumping you out, this looks like a perfect fit for you, but you can’t do anything about it, because we’re on hold and don't know when we’re going to get off of hold. That’s a little bit nerve-racking” (6)*  *“My last scan showed that my cancer progressed, and we were hoping to move me onto a trial. And yeah, they’re on pause right now. So right now, the next course of treatment would be one of the drugs on the trial and we're hoping that we can then make an appeal to my insurance company for a compassionate whatever that they would let me use the other drug that is showing promising results in the trial.” (6)* | | **X** |  |  | **F27** | |
| 2.The added burden due to cancer   - 1. Mental health and Stressors | *“not doing what they’re supposed to be doing” (6)*  *“And it’s just incredibly frustrating to feel like a lot of people aren’t taking it seriously. But for anything to actually change, and for us to move forward, everybody has to take it seriously. We’ve all been in the cancer boat, we know what it takes to adapt. Why can’t these other fools do that as well?” (6)*  *“It’s increased my anxiety quite a bit. It’s made me really scared to go out in public... Because I don’t look actively ill, so people don’t think that I have any kind of issues with my health. So, I don’t outwardly appear to have any kind of illness, which I think makes people think that I’m overreacting.” (6)*  *“I think people who haven’t been through cancer or who haven’t been compromised, I think they just have trouble having empathy for those of us who have experienced that. And so, they can’t see the severity of the situation. I’ve definitely seen that in a lot of my friends and family.” (6)* | | **X** |  |  | **F28** | |
| 2.The added burden due to cancer   - 1. Compounding uncertainty | *“When I was in treatment, I knew I was going to have eight treatments. It was going to take six months. I knew I would be hopefully done at the end of that. But with this, they said two weeks, and here we are four months later. So, there is not really a solid end. And the feeling of not knowing when you’re going to be able to...or if you’re going to be able to return to anything that you used to do has been very unsettling and very hard.” (7)*  *“We don’t know if there is other health issues that are going to come up, like if history [of cancer] is going to somehow make a COVID case worse or it would make me more susceptible.” (7)*  *“So every day is just kind of a, to me, it's kind of weighing my options. What’s worse, getting a recurrence or getting COVID?” (7)* | | **X** |  |  | **F29** | |
| 1. The unexpected advantages of a cancer history    1. Coping strategies | *“I’ve been trying to talk to a lot of friends and stay in touch with people. Because I think for me one of the hardest parts is the isolation piece. And struggling with depression and wanting to isolate on my own, and then being forced to, is its own different thing. And it’s really hard. And so, trying to fill my time, because I’m stuck at home all day, and just getting a schedule and trying to reach out to friends more often.” (7)*  *“I feel like I kind of reverted back to my old coping mechanisms for when I was immunocompromised, and I had to stay home. I literally still have all the old colouring books and everything that I used to have from then, and I was just doing them again because I didn’t know what else to do. And I was like, well, it worked then, so it might work now.” (7)* | | **X** |  |  | **F30** | |
| 3.The unexpected advantages of a cancer history  b. Resiliency | *“But for the first time I feel like other people who haven’t had cancer realize what the fear of getting sick is. Because I was scared about getting sick before this happened, just, it wasn't of COVID, it was of relapse. Well, now people who don’t have COVID are scared they're going to get COVID. And I feel like, for the first time, people are on my level.” (8)*  *“I feel like I have an advantage to some aspect on the mental game we’re playing. Because I know what it’s like. So I noticed, really when this all started, where people were like, ‘Oh, my gosh, we need masks. Oh my gosh, all this.’ Like, you guys are all freaking out. I’ve been doing this for four years. Like, I’m going to put a mask on and go to the store. Like, chill out. It’s not the end of the world. So, I feel like people are starting to understand what it’s like to not have a certain future. And everybody’s response to that is, to some extent, funny to me, because you guys all gave me crap about it for the last four years. And now you’re starting to be like, ‘Oh, wow, this is legit. Like, it’s scary.’ And it's like, ‘Yeah, it is.’ Shocker.” (8)* | | **X** |  |  | **F31** | |
| 1. AYA recommendations and resources | “I think for me, it’s just a simple phone call [from loved ones], simple phone calls go through a long way with me” (8)  “I don’t know if it’s necessarily just pandemic wise, but at least for me and others I’ve talked with, young adults really aren’t told about other young adult people or groups or things to do outside of getting sick. Like, I didn’t know anybody, and it was all on me to figure it out. But I walk in the hospital and there’s flyers for all, like, breast cancer groups and, you know, older adult groups. And it seems like the young adult community is something that isn’t talked about, isn’t looked at.” (8) | | **X** |  |  | **F32** | |
