# Supplementary material for: The unmet supportive care needs of people affected by cancer during the COVID-19 pandemic: an integrative review
Source: J Cancer Surviv. 2022 Oct 29;17(4):1036–56. doi: 10.1007/s11764-022-01275-z (PMC9616701; doi:10.1007/s11764-022-01275-z)
Supplement: Supplementary file 3 — Supplementary file3 (pdf 24 KB) [file 11764_2022_1275_MOESM3_ESM.docx]

**Supplementary Table 3 Quantitative Findings**

| **Author and Year** | **Physical Needs** | **Psychological/Emotional Needs** | **Cognitive Needs** | **Patient-Clinician Communication Needs** | **Health System/Information Needs** | **Spiritual Needs** | **Daily Living Needs** | **Interpersonal/intimacy Needs** | **Practical Needs** | **Family Related Needs** | **Social Needs** |
| --- | --- | --- | --- | --- | --- | --- | --- | --- | --- | --- | --- |
| Amidei C et al.,2020.  USA | 6% of patients were asked to complete DNRs when at this time point in the patient’s care they were “doing well”. | 92% reported generally greater anxiety.  69% feared being diagnosed with covid  27% reported increases or new emerges of mental health challenges  37% of patients had concerns that the hospital staff working where they were being treated didn’t have sufficient PPE | **Not reported** | 31% reported substantial interruption to contacting clinical teams regarding medical concerns | 71% of individuals in their brain tumour constituencies feared contracting COVID-19 while attending doctor visits or being hospitalised.  69% of brain tumour patient organisations said that in the communities they serve, patients and caregivers were concerned about treatments being delayed, cancelled, modified, or substituted because of the pandemic.  24% were redirected to a different treatment centre.  35% Challenges in participating in clinical trials/ participation being delayed or cancelled | 31% End of life issues- they did not report on the exact issues. | 22% had problems with the supply of medicine/ shortages  34% reported food shortages  16% had difficulties with food delivery or click and collect services at their local supermarket | **Not reported** | 37% reported interruptions to transportation due to COVID-19  12% had issues with medical insurance / reimbursement  25% experience loss of employment  37% had concerns about meeting financial commitments  (i.e., mortgage repayments, loans, insurances) | 49% reported an additional childcare pressure (i.e., home school) | 71% reported an increased pressure of self- isolation (i.e., visitor restrictions) |
| Büntzel J et al., 2020  Germany | “During the lockdown 127/342 (37.1%) participating patients have already registered minor physical wellbeing.” | 42.9% (150/350) of patients felt strong or very strong mental stress with this actual pandemic situation  119 patients reported about beginning mental stress.  57% (69/121) Hospitalised patients reported additional mental stress because of visitor restrictions. | **Not reported** | **Not reported** | 69.2% (247/357) of participants reported to be confused by the public discussion and information about COVID-19. | **Not reported** | **Not reported** | **Not Reported** | 38.5% (132/342) expected difficulties to get their individual therapies itself.  52.5% (186/354) were afraid of prolonged breaks between therapies or waiting times for necessary treatments because of the COVID-19 pandemic. | **Not Reported** | 67.3% (239/355) persons felt strongly or very strongly restricted by the official regulations.  73.1% (226/309) Out-patients/ cancer survivors reported distress because of isolation. |
| Caston N et al., 2021  USA | **Not reported** | 25% reported social distancing behaviours affected their mental health a lot.  39% reported as having more fear of COVID-19  Positive association between fear of COVID, symptoms of psychological distress and delay in receiving care. | **Not reported** | **Not reported** | **Not reported** | **Not reported** | **Not reported** | **Not reported** | 47% reported delaying any type of care due to COVID-19  (1% reported income loss  1% reported to insurance loss  27% Hospital or provider election  13% patient election  5% difficulty accessing medications or other medical care) | **Not reported** | 25% reported social distancing behaviours affected their mental health a lot. |
| Davis K et al., 2021  USA | 1% of “My Chart” responses reported fatigue  3% of telephone responses reported fatigue  1% Of “My Chart” responses Reported pain interference with activities of daily living  4% Of telephone responses Reported pain interference with activities of daily living  6% of “My Chart” responses reported deficits to physical function.  9% telephone responses reported deficits to physical function | 4% of “My Chart” responses reported anxiety  11% of telephone responses reported anxiety  5% of “My Chart” responses reported depression  6% of telephone responses reported depression  10% of “My Chart” responses reported practical or psychosocial needs  21% of telephone responses reported practical or psychosocial needs | **Not reported** | **Not reported** | **Not reported** | **Not reported** | 5% of “My Chart” responses reported nutritional needs  10% of telephone responses reported nutritional needs**.** | **Not reported** | **Not reported** | **Not reported** | **Not reported** |
| Dimelow J et al., 2021  United Kingdom, UK | **Not reported** | “Fears of getting the COVID-19 virus were slightly lower than fears of cancer recurrence in that 34 had little or no fears of the virus compared to 21 of having recurrence. Similar numbers (7 virus, 9 recurrence) either had a lot of fears or were fearful all the time, with 4 patients having significant fears for both and 12 patients with either or both.” | **Not reported** | **Not reported** | **Not reported** | **Not reported** | **Not reported** | **Not reported** | **Not reported** | **Not reported** | **Not reported** |
| Falcone R et al., 2020  Italy | **Not reported** | 65.70% of all patients reported fear/anxiety related to the COVID-19 pandemic.  Patients reported that the COVID-19 outbreak is having on their emotional state Little= 15.7%, some= 34.3%, quite a bit= 31.4% and very much 18.6%. | **Not reported** | **Not reported** | **Not reported** | **Not reported** | **Not reported** | **Not reported** | 55.7% of all patients reported feeling less medically protected due to the COVID-19 outbreak  41.4% reported feeling that their disease will be affected by the COVID-19 outbreak | **Not reported** | Impact of the COVID-19 pandemic on quality of life, little= 14.3%, some= 34.3%, quite a bit= 38.6% and very much= 12.9% |
| Fisher P. A et al 2021  USA | Over half of parents indicated that the COVID-19 pandemic negatively affected their physical well-being in the following areas: **Sleep 53.4%, Eating 52.3%, Exercise 53.4%** | 40.9% had difficulty sleeping when thinking about COVID-19  27.3% had temper outbursts thinking about COVID-19  22.7% were easily distressed when seeing something that reminds them of COVID-19  62.3% Parents also reported the pandemic negatively affected their mood  71.1% parents reported increased anxiety | **Not reported** | **Not reported** | **Not reported** | **Not reported** | **Not reported** | **Not reported** | Parents reported a mean of 7.52 disruptions to their lives. | 89.6% reported the closure of schools/childcare centres due to the pandemic was disrupting  68.8% reported an inability to visit or care for a family member was disruptive | 45.2% reported feeling less to much less socially connected  47.6% felt slightly less socially connected  93.8% reported the stay-at-home order due to the COVID-19 pandemic was disruptive |
| Hulbert- Williams J. Nicholas et al., 2021  United Kingdom | **Not reported** | More symptoms of anxiety, stress, depression, and poorer QOL following pandemic onset. | **Not reported** | High unmet needs in relation to care co-ordination and treatment decision making involvement, highlighting the broad reaching consequences stemming from the discouragement of active hospital attendance. | Four of the top 5 most increased patient needs related to hospital care and access, reflecting the consequences of reduced face to face hospital attendance in view of COVID-19 transmissions. care | **Not reported** | **Not reported** | Clinically significant changes to sexual feelings, sexual relationships, and information regarding sexual relationships. | **Not reported** | Positive association between unmet needs in patients whose support networks focus on family communication, practical caring tasks and accessing their own support.  Reported additional support in coping with the demands of home- based caring pressures.  Significant change to the opportunities to participate in decision making about the persons cancer treatment, communication with the family, and caring for the person with cancer. | The most significant domain of unmet needs relates to work and social settings. |
| McFarlene. P et al., 2022  United Kingdom | 30% of patients reported pain  47% of patients reported shortness of breath  75% of patients reported weakness of lack of energy  20% of patients reported nausea  12% of patients reported vomiting  37% of patients reported poor appetite  15% of patients reported constipation  10% of patients reported sore or dry mouth  35% of patients reported feeling drowsy  65% of patients reported having poor mobility | 50% of patients reported feeling anxious or worried  27% of patients reported feeling depressed | **Not reported** | **Not reported** | 5% felt they had received as much information as they wanted to | 37% of patients reported feeling at peace | **Not reported** | **Not reported** | 27% had practical issues from their illness. | 75% of patients reported their family and or friends felt anxious or worried  30% of patients reported feeling able to share feelings with family and friends | **Not reported** |
| Shay. L Aubree et al., 2021  USA | 39% reported being less physically active | 90% reported anxiety due to worry about their health or their family’s health  46% are worried about their job security  36% reported having less sleep  18% reported sleeping more | **Not reported** | 36% reported scheduling delays in relation to treatment  28% reported delayed or reduced communication with my healthcare team | 28% reported reduced access to treatment services | **Not reported** | 7% reported trouble getting food and other personal necessities  15% reported trouble getting their medications | **Not reported** | 36% reported decreased productivity at work  28% reported a reduction to job hours or wages  18% reported being temporarily laid off from work  8% reported being forced to use vacation of sick time  5% reported loss of employment | **Not reported** | 64% reported feeling more isolated |
| Kabak. V. et al., 2020  Turkey | 20.8% reported feeling unwell a lot of the time  24.5% reported pain | 18.9% reported feeling down or depressed  19.8% reported lack of energy/ tiredness  20.8% reported feelings of sadness  26.4% reported anxiety  46.2% reported worry about the results of treatment are beyond their control  48.1% reported fear about the cancer spreading  51.9% reported uncertainty about the future | **Not reported** | **Not reported** | **Not reported** | **Not reported** | **Not reported** | **Not reported** | 36.8% reported not being able to do the things they used to do | **Not reported** | **Not reported** |
| Ostermann. C et. Al ., 2020  USA | 15% reported uncontrolled symptoms | 6% had a distress score of greater than or equal to 8/10 | **Not reported** | 13% reported medication issues (i.e., refills, questions, compliance, cost)  10% reported feeling uncertain about how to contact the care team  7% reported a question about the plan of care  7% reported being unaware of the next oncology appointment  3% had no future appointment scheduled or schedule is incorrect | 13% reported an interest in advance directives or healthcare power of attorney | **Not reported** | **Not reported** | **Not reported** | 17% reported financial difficulties | **Not reported** | **Not reported** |
| Zomerdijk. N et al., 2021  Australia | **Not reported** | Psychological distress and concern about impact of COVID-19 on cancer management were significant predictors of fear of cancer recurrence. (*Β*- 0.28 *p=0.001*) and *Β* - 0.25 *p=0.004*)  Unmet supportive care needs was a significant predictor of psychological distress (*B- 0.27 p=0.000 )*  Positive association between psychological distress and unmet needs. (Β- 0.27 p=0.000) | **Not reported** | **Not reported** | Concern about impact of COVID-19 on cancer management was a significant predictor of psychological distress (B- 0.09 p=0.096) | **Not reported** | **Not reported** | **Not reported** | Financial concerns were a significant predictor of psychological distress ***(****B=0.20 p=0.000)*  Lost income was a significant predictor of unmet supportive care needs (*B- 0.13 p=0.009)* | Limited opportunity for family support was a significant predictor of psychological distress (*B- 0.08 p=0.077)* | **Not reported** |
